# Breaking the Aromaticity Trap: *N*‑Silylation-Induced Formation of Stable 2,3-Dihydro-4-dialkylaminopyridin-1-iums

**DOI:** 10.1021/acs.joc.6c00105

**Published:** 2026-06-25

**Authors:** Valery A. Verkhov, Artyom A. Yakubenko, Benjamin Begović, Elena Yu. Tupikina, Alexander S. Antonov

**Affiliations:** 1 St. Petersburg State University, Institute of Chemistry, Universitetskii pr. 26, 198504 St. Petersburg, Russian Federation; 2 Institute of Organic Chemistry, University of Regensburg, D-93053 Regensburg, Germany

## Abstract

The reactivity of pyridines toward organolithium reagents is dominated by the nucleophilic addition to the C2 position, leading to the formation of labile 1,2-dihydropyridines that readily rearomatize. Herein, we report an unexpected deviation from this classical pathway. Trimethylsilylation of 4-dialkylaminopyridine steers their reaction with alkyl lithium reagents (*n*-BuLi, *s*-BuLi, *t*-BuLi) toward the formation of 2,3-dihydro-4-dialkylaminopyridin-1-iums, isolated as air-stable triflates, which are remarkably resistant to oxidation and deprotonation. This stability is attributed to pronounced orbital interactions and charge delocalization between the NR_2_ group and the pyridinium core creating a vinylogous amidine structural motif. At the same time, triisopropylsilylation completely suppresses nucleophilic addition, enabling the first case of room-temperature C2 lithiation of the pyridine core. This difference in the reaction pathway originated from a dramatic decrease of the relative steric accessibility of C2(6) pyridine carbon atoms.

## Graphical abstract

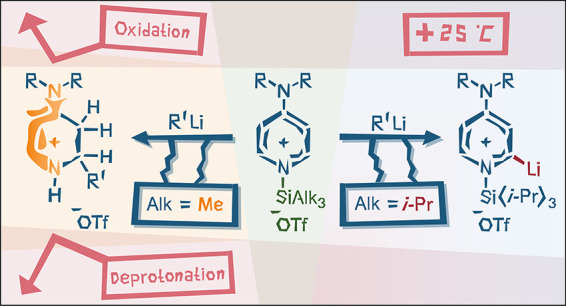

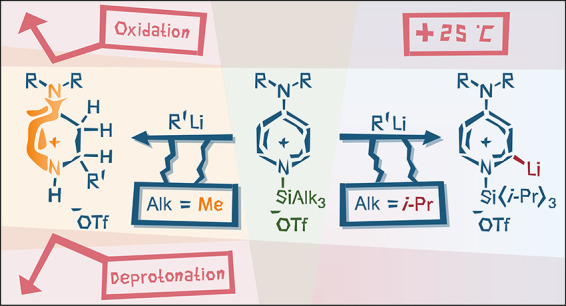

## Introduction

Dihydropyridines (DHPs), the partially hydrogenated analogues of pyridines, are a class of heterocyclic compounds of substantial importance in fields ranging from pharmaceuticals and agrochemicals to materials science.^1–3^ Among them, 1,4-dihydropyridines (1,4-DHPs) have garnered the most significant attention due to their potent biological activities, most notably their calcium channel blocking properties, which led to the development of widely prescribed antihypertensive drugs such as nifedipine and amlodipine.^4–8^ The classical and most prevalent method for the synthesis of stable 1,4-DHPs, typically stabilized by electron-withdrawing substituents, is the Hantzsch multicomponent condensation.^9,10^

Nucleophilic addition of organometallic reagents to the pyridine nucleus constitutes a method for the generation of 1,2- and, in a specific case, 1,4-dihydropyridines (1,2-DHPs and 1,4-DHPs). For unsubstituted pyridines, this reaction proceeds preferentially via addition to the C2 position, yielding 1,2-DHP intermediates. However, these species are notoriously unstable and undergo rapid rearomatization upon exposure to air, typically resulting in the formation of 2-substituted pyridines as the final products (Scheme 1a).^11^ Despite this inherent instability, which has limited their isolation and practical application, 1,2-DHPs have been identified as key intermediates in various processes, notably in hydride-transfer catalysis.^12^

**Scheme 1:**
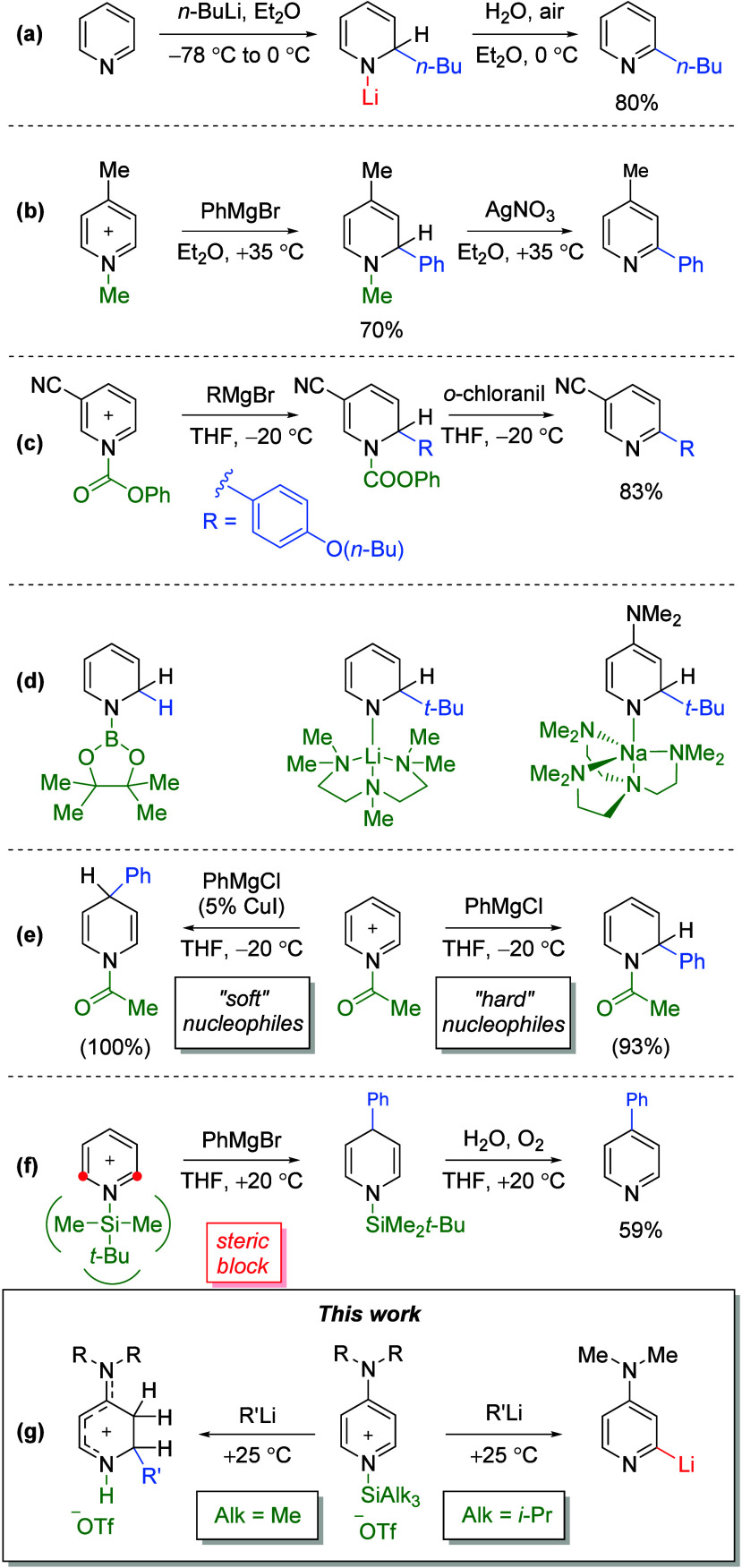
Reactivity of Pyridines toward Organometallic Reagents^a^

a (a) Formation of unstable 1,2-DHPs from unsubstituted pyridines and their subsequent rearomatization; (b) stabilization of 1,2-DHPs by N1-alkylation and their rearomatization triggered by AgNO_3_; (c) stabilization by a bulky N1-acyl group (e.g., COOPh) and oxidative rearomatization with *o*-chloranil; (d) stabilization via Bpin incorporation in catalytic cycles or by alkali metal cation coordination; (e) regioselectivity control using hard vs soft nucleophiles; (f) steering nucleophilic addition to C4 by N1-silylation with bulky trialkylsilyl groups; (g) this work: unprecedented formation of stable 2,3-dihydropyridin-1-ium salts via the reaction of N1-TMS 4-dialkylaminopyridines with organolithium reagents, contrasting with conventional C2 metalation observed for the N1-TIPS DMAP analogue.

To circumvent this inherent instability of 1,2-DHPs, several stabilization strategies have been developed. A common approach involves quaternization of the pyridine nitrogen (Scheme 1). While alkyl groups can afford isolable 1,2-DHPs, these systems often remain susceptible to rearomatization, for instance, upon treatment with silver nitrate (Scheme 1b).^13^ Employing a more sterically defined and electron-withdrawing group, such as COOPh, can yield stabilized 1,2-DHPs that rearomatize only under the action of strong oxidants like *o*-chloranil (Scheme 1c).^14^ Further methodologies include the incorporation of a Bpin group or coordination to alkali metal cations, which effectively blocks the rearomatization pathway and allows for the isolation of stable complexes (Scheme 1d).^12,15,16^ The regioselectivity of nucleophilic addition is highly dependent on the character of the organometallic reagent. The use of ″hard″ nucleophiles (e.g., Grignard reagents) typically favors addition at the C2 position, yielding 1,2-DHPs, whereas ″soft″ nucleophiles (e.g., organocopper reagents) favor addition at C4, resulting in 1,4-DHPs (Scheme 1e).^17^ Furthermore, the reaction pathway can be altered by employing bulky trialkylsilyl substituents on the pyridine nitrogen. These groups sterically block the C2 position, thereby directing nucleophilic attack to the C4 position (Scheme 1f).^18,19^

Much less is known about 2,3-DHPs, which are the most unstable members of the dihydropyridine family. They are reported as reactive intermediates, but to the best of our knowledge, only perfluorinated 2,3-dihydropyridine was isolated.^20,21^

While most known stabilized 1,2-, 1,4-, and 2,3-dihydropyridines rely on electron-poor pyridinium frameworks, an intriguing and largely unexplored question is whether strongly electron-rich pyridines can be driven into similarly stabilized nonaromatic states by an appropriate choice of the 1-substituent.

The reactivity of pyridines containing strong electron-donating groups, particularly 4-dimethylaminopyridine (DMAP)—a cornerstone reagent in organic synthesis renowned for its superior nucleophilic catalysis in acyl transfer reactions and its versatility as a ligand—remains largely unexplored in reactions with organolithium reagents.^22–29^ The strong electron-donating effect of the 4-dimethylamino group creates a unique electronic environment, making the outcome of its interaction with highly reactive organolithium compounds particularly challenging to predict. In this context, 1-silylation of DMAP is particularly appealing as a modular handle to tune both the electronic structure and steric environment at the pyridine nitrogen, yet its consequences for the fundamental reactivity landscape of the pyridine ring have remained largely unexplored.

We therefore set out to establish a mechanistic framework that links strong conjugation of the 4-NR_2_ group with the pyridine core additionally facilitated by 1-silylation to the accessibility and stability of nonclassical dihydropyridinium states. Such a framework is expected to be broadly useful for the rational design of pyridine functionalization strategies. Following this concept, we investigate the reaction of 1-trimethylsilylated 4-dimethylaminopyridine (Me_3_Si-DMAP) and related 4-dialkylaminopyridines with organolithium reagents and report the formation of previously unknown extremely stable 2,3-dihydropyridin-1-iums (Scheme 1g, right). The stability of these novel compounds is demonstrated by their isolation in air and inertness toward oxidants such as silver nitrate. A crucial finding is the dependence of the reaction pathway on the steric demand of the 1-silyl group. Remarkably, replacing the Me_3_Si group with a triisopropylsilyl (*i*-Pr_3_Si) group completely suppresses the nucleophilic addition and facilitates selective C2–H dispersion-directed deprotonation,^30^ thus manifesting the first successful metalation of the pyridine core at room temperature (Scheme 1g, left).

## Results and Discussion

### 1 Regio- and Type-Selectivity of Nucleophilic Addition of Organolithiums to DMAP

Keeping the possibility of trimethylsilyl groups to redirect the organometallic reagent to C3 (in the case of metalation)^30^ and C4 (in the case of nucleophilic addition)^18,19^ positions in mind, we started this work with the prediction of the regioselectivity of nucleophilic addition of organolithiums to DMAP. We analyzed the molecular electrostatic potential (ESP) mapped onto the electron density isosurface (ρ = 0.001 au) of the studied systems (Figure 1a; see also Table S1 in the SI). For DMAP itself, the ESP values at the carbon atoms of the pyridine ring are predominantly negative, reflecting the electron-rich π-system imparted by the electron-donating dimethylamino group, which reduces the electrophilic character of the ring carbons and, consequently, their susceptibility to nucleophilic aromatic substitution. Furthermore, the ESP becomes increasingly negative on carbon atoms closer to the nitrogen atom.

**Figure 1:**
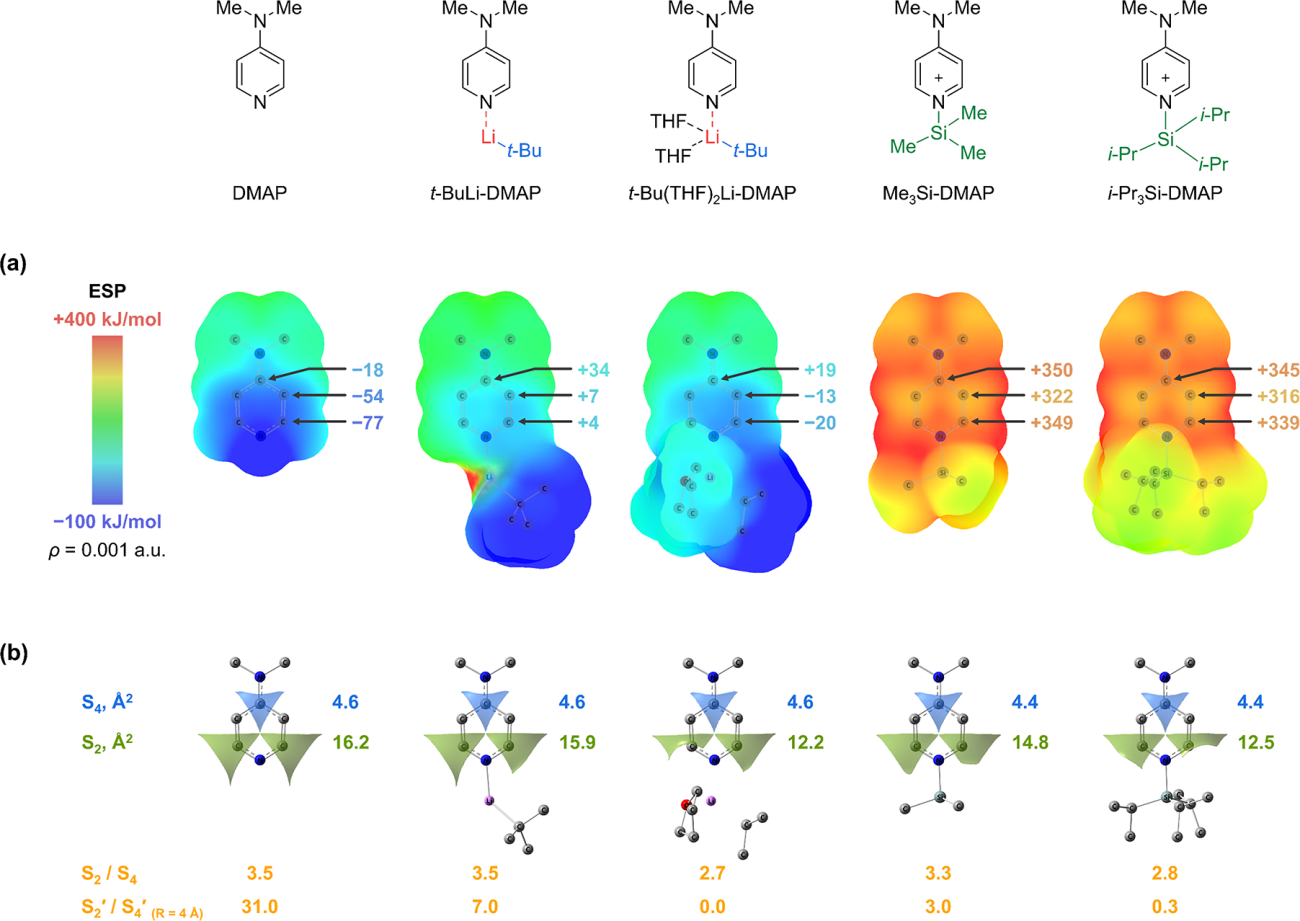
(a) Molecular electrostatic potential (ESP, kJ/mol) mapped onto the 0.001 au electron density isosurface. Blue regions indicate negative ESP values; red regions indicate positive ESP values. (b) Analysis of steric accessibility. Accessible surface areas of pyridine carbon atoms, acquired by the intersection of the electron density (ED) isosurface of 0.001 au and the ED basins of the corresponding atoms. Green surfaces and values represent C2/C6 atomic basins; blue surfaces and values represent C4 atomic basins. The relative ratio of these accessible surface areas (S_2_/S_4_, yellow values) is shown. Relative steric accessibility (S_2_′/S_4_′, yellow values) determined using a rolling sphere method (*R* = 4 Å) on the van der Waals surface. Calculations performed at the PW6B95-D3­(BJ)/def2-TZVPD (CPCM = THF) level of theory. Hydrogen atoms are omitted for clarity.

Coordination of an organolithium reagent to the pyridine nitrogen atom reduces the electron density in the ring, leading to an increase in the ESP at the carbon atoms. This coordination makes the subsequent nucleophilic attack an intramolecular process, which explains its ease despite the noticeably negative ESP values (Figure 2; see also Figure S1 in the SI).

**Figure 2:**
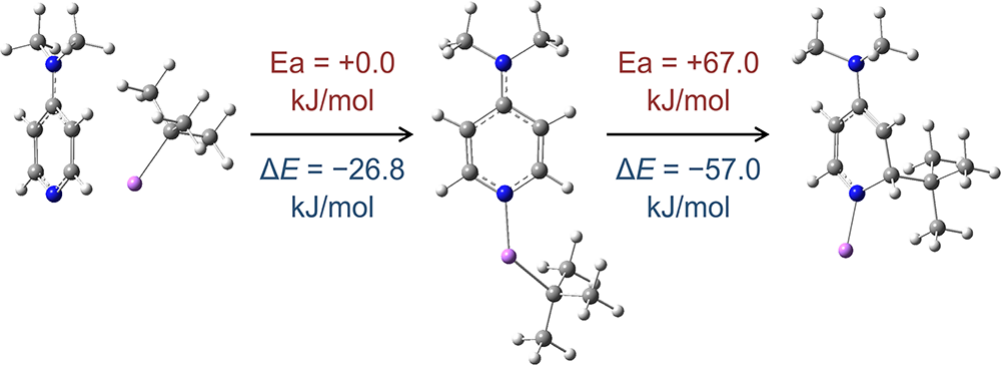
Intramolecular nucleophilic addition pathway via lithium coordination. Potential energy scan along the C2­(DMAP)–C­(*t*-Bu) reaction coordinate. The pathway involves barrierless coordination of *t*-BuLi to the pyridinic nitrogen followed by intramolecular nucleophilic attack at C2. Δ*E* values represent energy differences between potential energy surface minima; *E*_a_ denotes the activation energy barrier between them. Calculations performed at the B3LYP-D3­(BJ)/6–311++G­(d,p) level with CPCM­(THF) solvation.

Quaternization of the nitrogen with a trialkylsilyl group (Alk_3_Si) induces a significant outflow of electron density from the ring, resulting in strongly positive ESP values at the carbon atoms, thereby activating them toward nucleophilic attack. Notably, the trimethylsilyl (Me_3_Si) group induces a more pronounced increase in ESP compared to the triisopropylsilyl (*i*-Pr_3_Si) group. The highest ESP values are localized at the carbon atoms in positions 2, 4, and 6, which predict the most probable sites for intermolecular nucleophilic addition.

Next, the steric accessibility of positions C2, C4, and C6 of trialkylsilylated DMAPs as potential sites for nucleophilic attack was evaluated (Figure 1b; see also Table S2 in the SI). This assessment was based on the accessible surface area of the atomic basin for each carbon atom on the electron density isosurface (ρ = 0.001 au), with basin boundaries defined by the condition of zero flux in the electron density gradient.^30^ For a comparative analysis, the relative steric accessibility of the C2 and C6 positions to the C4 position was expressed as the S_2_/S_4_ ratio. Introduction of a substituent at the N1 position reduces the accessible surface area at the C2 and C6 positions. A comparison of silyl groups demonstrates that the bulky triisopropylsilyl group in *i*-Pr_3_Si-DMAP (S_2_/S_4_ = 2.8) provides significantly greater steric shielding of the C2 and C6 positions than the trimethylsilyl group in Me_3_Si-DMAP (S_2_/S_4_ = 3.3), as reflected by the lower accessibility ratio for the *i*-Pr_3_Si derivative. Despite this differential shielding, nucleophilic attack remains sterically more favored at the C2 and C6 positions than at the C4 position across all systems studied.

However, accounting for the effective size of the organolithium reagent using a rolling sphere method across the van der Waals surface of the 1-silylated systems revealed that with a sphere radius (R) of 4 Å, the accessibility of the C2 and C6 positions in *i*-Pr_3_Si-DMAP approaches zero (S_2_′/S_4_′ = 0.3). This contrasts sharply with the 1-trimethylsilylated system (S_2_′/S_4_′ = 3.0) under the same conditions (Figure 1b; see also Figure S2 in the SI).

Finally, the relative stabilities of nucleophilic addition products at different positions were evaluated (Figure 3). The loss of aromaticity during nucleophilic addition allows the ring to adopt nonplanar conformations. For the addition at the C2 position, steric repulsion between the nucleophile and the N1 substituent induces ring puckering, which spatially separates these bulky groups and lowers the energy. In contrast, C4 addition places two substituents at the same carbon atom, creating steric strain that cannot be relieved through conformational changes; consequently, C2 addition products are thermodynamically more stable than their C4 counterparts. Expectedly, the bigger the nucleophile is, the lower the stability of the C4 adduct is (see Figure S3 in Supporting Information).

**Figure 3:**
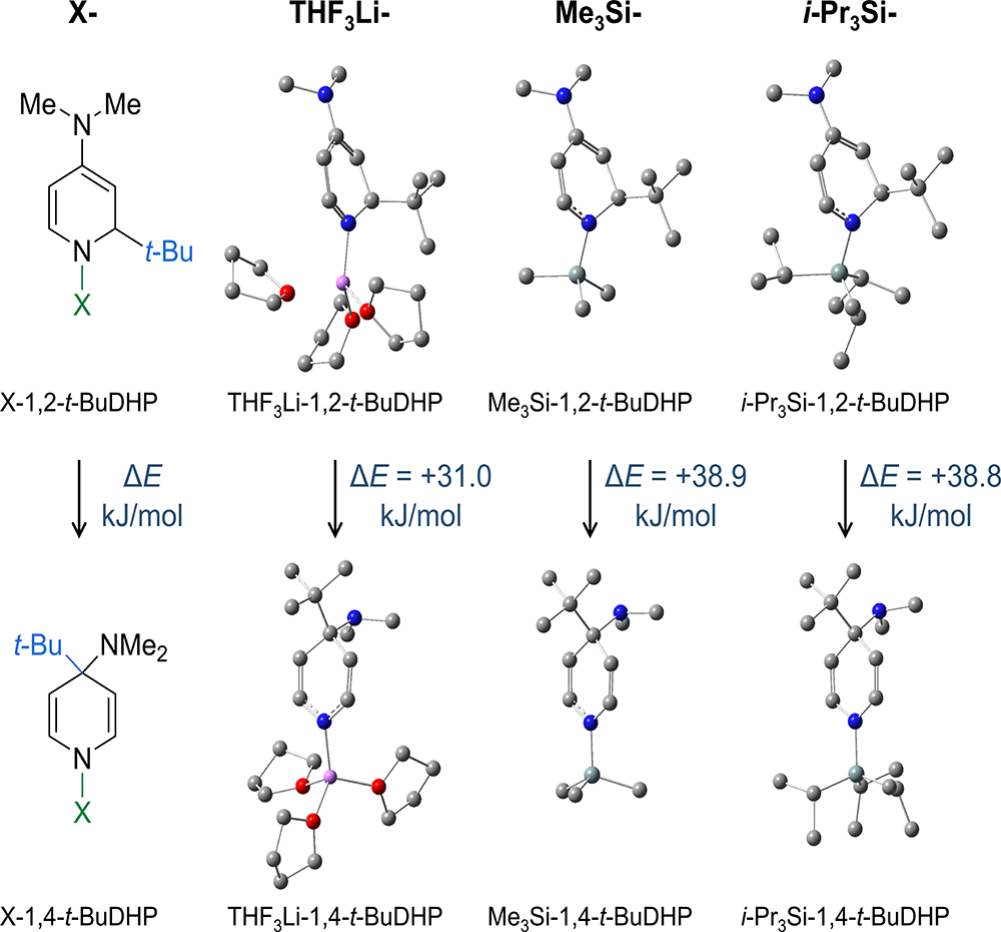
Relative stability of C2 vs C4 nucleophilic addition products. Top row: C2-addition products; bottom row: C4-addition products. Relative electronic energies (Δ*E*, kJ/mol) between corresponding C2 and C4 adducts are indicated. From left to right: general structures with substituent X at N1 position; THF_3_Li-substituted; Me_3_Si-substituted; and *i*-Pr_3_Si-substituted derivatives. The consistent energy preference for C2-addition products demonstrates their superior thermodynamic stability across all substituents. PW6B95-D3­(BJ)/def2-TZVPD (CPCM = THF). Hydrogen atoms are omitted for clarity.

The energy difference between C2 and C4 addition pathways increases when changing from X = THF_3_Li (Δ*E* = +32 kJ/mol) to X = Alk_3_Si (Δ*E* = +39 kJ/mol). This enhancement results from orbital interaction between the dimethylamino and trialkylsilyl groups. In C4 adducts, this conjugation is disrupted as the dimethylamino group is forced to twist out of plane, increasing the total electronic energy. For C2 addition, where bulky groups are effectively separated, the nature of the alkyl group (Me_3_Si vs *i*-Pr_3_Si) shows negligible influence on thermodynamic stability.

In summary, the reaction of organolithium reagents with DMAP proceeds through an intramolecular pathway involving the initial lithium coordination to the pyridine nitrogen atom. In contrast, for N1-silylated DMAPs, nucleophilic addition is an intermolecular process. C2 addition is thermodynamically favored over C4 addition due to the ring puckering, which alleviates steric strain by spatially separating bulky substituents. However, for the *i*-Pr_3_Si-substituted system, the approach of the organolithium reagent is sterically hindered. This suppression of the nucleophilic addition pathway creates the possibility that alternative reactivities can become operative.

The experimental investigation was started by examining the nucleophilic addition of *n*-BuLi to DMAP in diethyl ether at room temperature (Scheme 2a). Following aqueous workup, the reaction yielded the expected C2-addition product **1a**. In contrast, the reaction of N1-trimethylsilylated DMAP led to the unexpected formation of stable 2,3-dihydropyridin-1-ium triflate **2a**·OTf together with a small amount of pyridone **3a** (Scheme 2b).

**Scheme 2:**
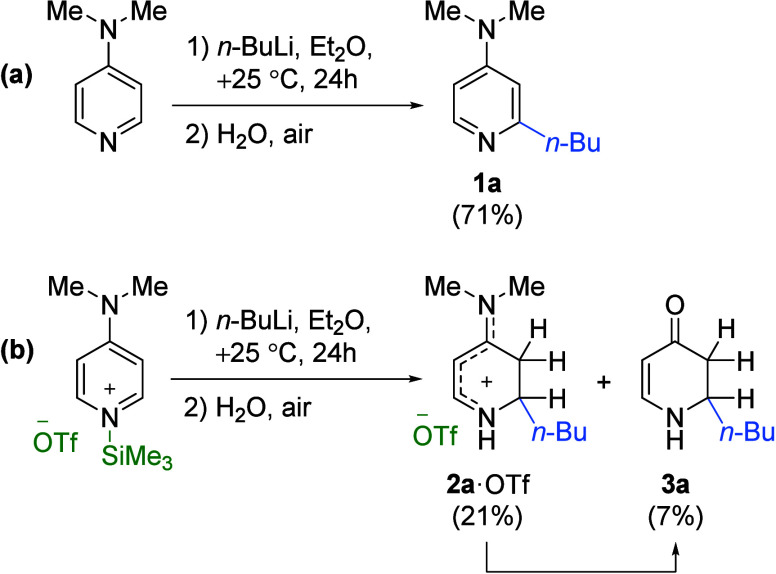
Comparison of Reaction Pathways for DMAP and Its N1-Trimethylsilylated Derivative with *n*-BuLi^a^

a (a) Conventional nucleophilic addition to DMAP yields C2-addition product **1a** after hydrolysis. (b) Reaction of Me_3_Si-DMAP leads to the unexpected formation of stable 2,3-dihydropyridin-1-ium triflate **2a**·OTf, accompanied by pyridone **3a** as a minor byproduct.

Optimal reaction conditions were carefully selected for each tested organolithium reagent to maximize nucleophilicity while minimizing decomposition via reaction with solvent, taking into account the known aggregation states of these reagents in solution (Table 1, see also Table S3 in the Supporting Information). Room temperature proved optimal in all successful cases. Diethyl ether was the solvent of choice for *n*-BuLi (run 3). For the more basic *sec*- and *tert*-butyllithiums, which react with diethyl ether at room temperature,^31^ a less CH-acidic benzene provided superior results (runs 5, 7). Consequently, 2,3-dihydropyridin-1-iums **2b**·OTf (R = *s*-Bu) and **2c**·OTf (R = *t*-Bu) were successfully synthesized. Single crystals suitable for X-ray diffraction analysis were grown for both compounds, enabling the unambiguous determination of their molecular structures (Figure 4).

**Figure 4:**
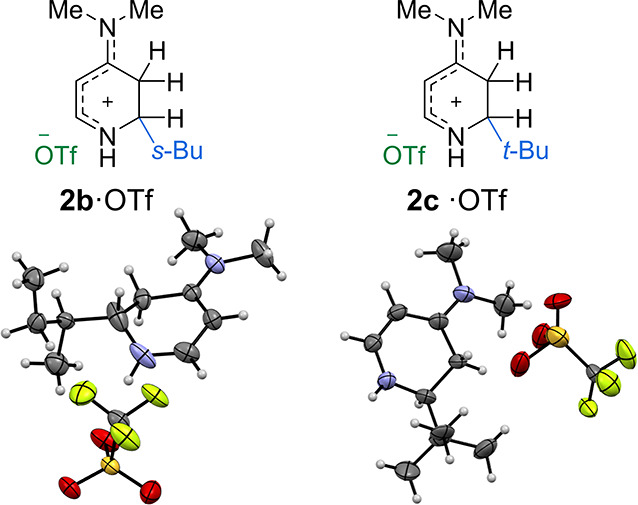
Solid-state molecular structures of salt **2b**·OTf (*s*-Bu) and salt **2c**·OTf (*t*-Bu), determined by single-crystal X-ray diffraction (50% probability level).

**Table 1:**
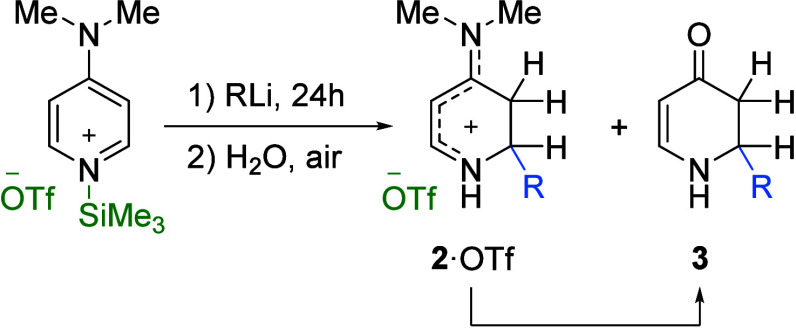
Reaction of Me_3_Si-DMAP with Organolithium Reagents

				product ratio		
run	RLi	solvent	*t*, °C	**2**·OTf	3	DMAP
1	*n*-BuLi	*n*-hexane	+25			1
2	*n*-BuLi	benzene	+25	0.09		1
3	*n*-BuLi	Et_2_O	+25	0.30	0.10	1
4	*n*-BuLi	THF	+25	0.11		1
5	*s*-BuLi	benzene	+25	0.35		1
6	*s*-BuLi	Et_2_O	+25	0.30		1
7	*t*-BuLi	benzene	+25	0.54		1
8	*t*-BuLi	Et_2_O	+25	0.20		1
9	EtLi	Et_2_O	+25	<0.01		1
10	Me_3_SiCH_2_Li	Et_2_O	+25	<0.01		1
11	MeLi	Et_2_O	+25			1
12	MeLi	THF	+50		0.03	1
13	PhLi	Et_2_O	+25			1
14	PhLi	THF	+50		0.01	1

Notably, Me_3_Si-DMAP remained completely inert to methyllithium, ethyllithium, trimethylsilylmethyllithium, and phenyllithium under all tested conditions, likely due to the higher aggregation (and as a result, lower nucleophilicity) of these reagents.^32^ For instance, even at 50 °C in THF, only traces of the products were detected (runs 12, 14).

Counterintuitively, increasing the amount of the organolithium reagent from 1 to 2 equiv of *n*-BuLi did not improve the conversion to **2a**·OTf but instead decreased selectivity. Under these conditions, the conventional C2-addition product **1a** was formed alongside dihydropyridin-1-ium **2a**·OTf and pyridone **3a**. This outcome is rationalized by the cleavage of the Me_3_Si group by the excess organolithium reagent, followed by the reaction of the generated DMAP with *n*-BuLi via the conventional pathway.

Replacing the Me_3_Si group with the more sterically demanding (*i*-Pr)_3_Si group led to a significant shift in the reactivity of DMAP. Upon treatment of (*i*-Pr)_3_Si-DMAP with *n*-BuLi in diethyl ether at room temperature and subsequent quenching with water in air, the major product was compound **4** (Scheme 3). The 2,3-dihydropyridin-1-ium salt **2a**·OTf and pyridone **3a** were formed only in minor amounts. The suppressed nucleophilic addition is attributed to the low steric accessibility of the C2 position. This steric blockade instead enables a metalation pathway to dominate. The initially formed 2-lithio derivatives undergo a 1,2-silyl migration (sigmatropic rearrangement) to afford the final product **4**, wherein the silyl group migrates from nitrogen to the adjacent C2-lithio carbon center. Notably, this represents the first reported example of a room-temperature metalation of pyridine.

**Scheme 3:**
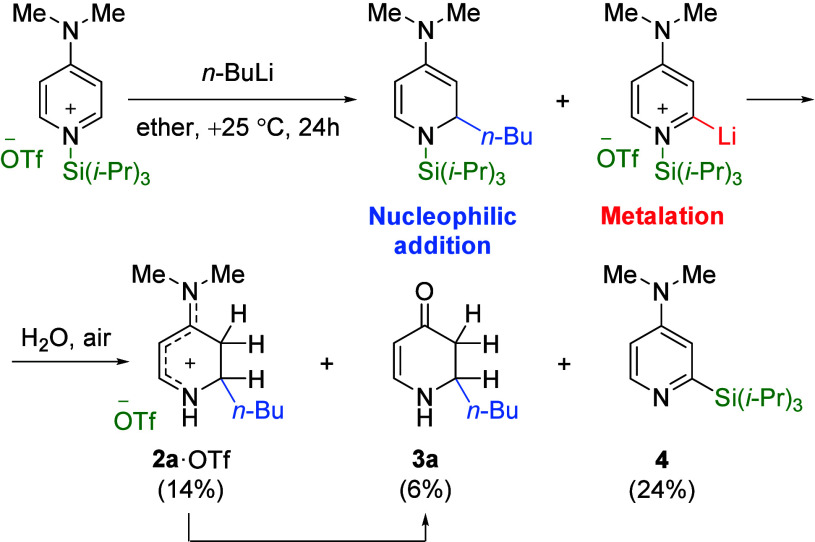
Reaction Pathway of (*i*-Pr)_3_Si-DMAP with *n*-BuLi^a^

a Reaction with *n*-BuLi yields an intermediate from the nucleophilic addition at C2, alongside a competing C2-lithiated intermediate from metalation. Subsequent hydrolysis leads to protonation of the addition intermediate, forming salt **2a**·OTf, which may undergo oxidation to pyridone **3a**. The lithiated intermediate undergoes a 1,2-silyl group transfer to afford the C2-substituted product **4**.

Subsequently, the influence of the nucleophile on the reaction outcome was evaluated under the identical conditions (Table 2). As the steric bulk of the nucleophile increases (from *n*-Bu to *s*-Bu and *t*-Bu), the nucleophilic addition pathway becomes progressively suppressed, facilitating the metalation reaction.

**Table 2:**
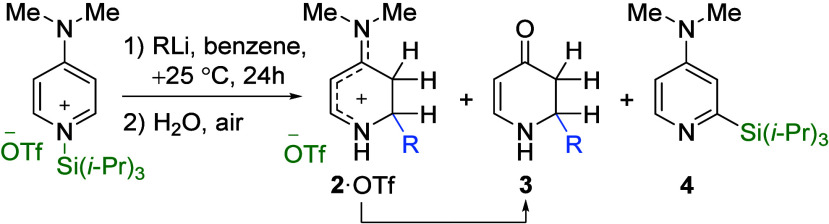
Reaction of (*i*-Pr)_3_Si-DMAP with Organolithium Reagents

		product ratio			
run	RLi	**2**·OTf	3	4	DMAP
1	*n*-BuLi	0.24	0.10	0.43	1
2	*s*-BuLi	0.05		0.08	1
3	*t*-BuLi	0.05		0.25	1

Overall, the introduction of an Alk_3_Si group to the position N1 of DMAP suppresses the efficiency of nucleophilic addition of organolithium reagent (up to the complete suppression in favor of metalation reaction in the case of the (*i*-Pr)_3_Si group) but does not affect the regioselectivity: for all tested reactions, addition occurs exclusively at position C2.

### 2 Stabilization of 4-Dialkylamino-2,3-dihydropyridiniums

All obtained dihydropyridiniums **2**·OTf demonstrated exceptional stability under ambient conditions, showing no signs of rearomatization to **1**. Moreover, **2**·OTf exhibited remarkable resilience toward oxidation, remaining unchanged upon treatment with silver nitrate. Furthermore, attempts to deprotonate the pyridinium nitrogen of **2**·OTf using strong bases—including ammonia, sodium hydroxide, or even proton sponge (p*K*_a_ = 12.1, water)—were unsuccessful. For instance, no characteristic H-bonded proton signal in the 17–19 ppm region^33^ was detected in the ^1^H NMR spectrum of the mixture of **2a**·OTf with an excess of proton sponge, indicating that no proton transfer occurred (Figure 5). We believe that this unusual feature for dihydropyridines originates from the presence of vinylogous amidine moiety in **2**,^34,35^ where one nitrogen donates electron density while the other can stabilize polarization, thus drastically increasing basicity.

**Figure 5:**
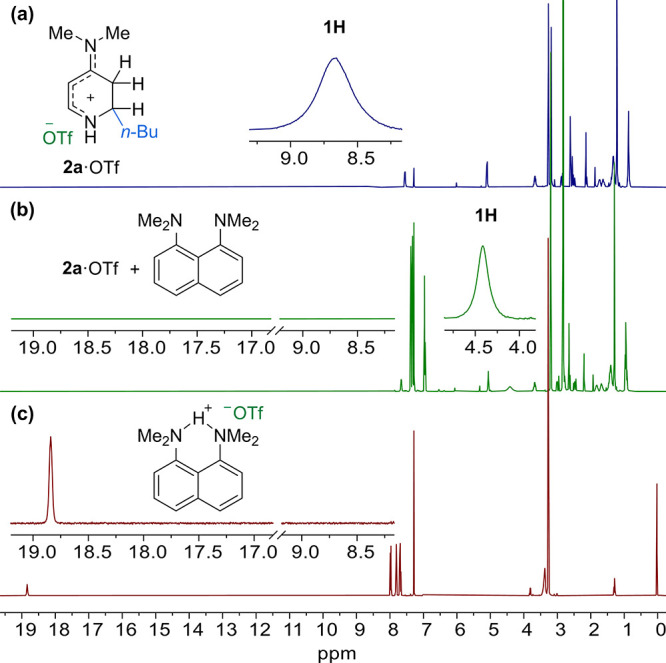
^1^H NMR (CDCl_3_, r.t.) spectroscopic evidence for the stability of salt **2a**·OTf. (a) Spectrum acquired in air. (b) Spectrum after addition of proton sponge, showing the absence of a signal in the 17–19 ppm region. (c) Reference spectrum of proton sponge after addition of HOTf, displaying the characteristic signal at 17–19 ppm corresponding to the proton located within the proton sponge’s internal basic cavity.

This collective evidence confirms the unusual stability of salt **2a**·OTf against both rearomatization and deprotonation, implying significant charge delocalization within the molecule, strongly preserving 2,3-dihydropyridine backbone. This is rationalized by orbital interaction between the dimethylamino group and the ring’s nitrogen atom. NBO analysis of salt **2c**·OTf (R = *t*-Bu) revealed significant orbital interactions as quantified by second-order perturbation theory. A high stabilization energy of 346 kJ/mol was calculated for the donor–acceptor interaction (*E* ^(2)^) between the lone pair of the dimethylamino nitrogen and the antibonding orbital of the C4–C5 bond, indicating pronounced orbital delocalization (Figure 6). A further substantial interaction (231 kJ/mol) was identified between the bonding orbital (BD) of the C4–C5 bond and the antibonding orbital (BD*) of the C6–N1 bond.

**Figure 6:**
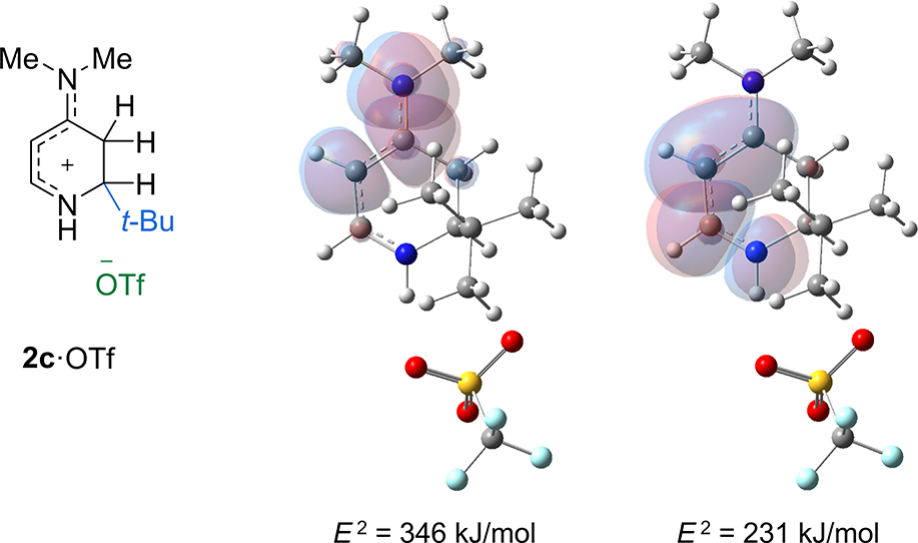
NBO analysis of dihydropyridin-1-ium salt **2c**·OTf illustrating key orbital interactions with high second-order perturbation theory stabilization energies (*E* ^2^). Values shown for LP­(NMe_2_)→BD*­(C4–C5) and BD­(C4–C5)→BD*­(C6–N1) interactions. Isosurface value for NBOs = ±0.02 au. Calculations performed at the PW6B95-D3­(BJ)/def2-TZVPD level with the CPCM­(THF) solvation model.

It was discovered that 2,3-dihydropyridin-1-iums **2** could also be generated by adding strong acids during the workup stage, following the nucleophilic addition of organolithium reagents to DMAP itself (Table 3). Interestingly, the nature of the acid plays a crucial role in the stabilization of **2**. Trifluoromethanesulfonic acid (HOTf) provided the highest yield of **2a** (run 1). However, the acid strength alone does not dictate the outcome. For instance, weak acetic acid (run 4) afforded a small amount of **2a**, whereas strong HCl (run 7) yielded none.

**Table 3:**
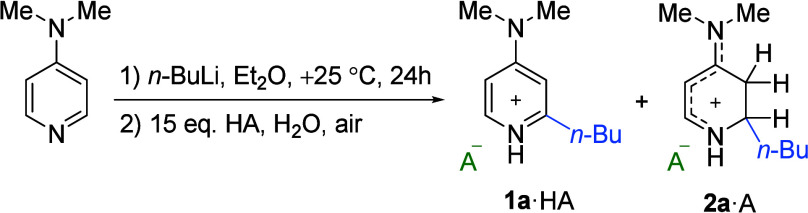
Formation of 2,3-Dihydropyridin-1-iums via Acidic Quenching

		product ratio		
run	HA	**1a**·HA	**2a**·A	DMAP
1	HOTf	1	0.77	0.25
2	CF_3_COOH	1	0.21	0.10
3	HBF_4_	1	0.19	0.10
4	CH_3_COOH	1	0.04	0.22
5	HClO_4_	1	0.03	0.28
6	HI	1	<0.01	0.08
7	HCl	1		0.06
8	HNO_3_	1		0.04
9	HBr	1		0.02
10	H_2_SO_4_	1		

The marked difference in efficacy between HOTf (highly lipophilic, weakly coordinating anion) and HCl (strongly coordinating chloride) suggests that the anion nucleophilicity and coordination ability play a crucial role. The noncoordinating nature of the triflate anion allows for effective charge delocalization and stabilization of the 2,3-dihydropyridin-1-ium cation, whereas more nucleophilic anions (such as Cl^–^) may promote alternative pathways or competitive rearomatization through ion-pair interactions.

In contrast to the N1-silylation strategy, the acid-quench approach provides inferior **2a**:**1a** selectivity. The reason lies in a different reaction pathway provided by N1-silylation. Thus, the reaction of N1-trimethylsilyl-DMAP with, for example, *n*-BuLi naturally leads to the formation of intermediate 1,2-dihydropyridine **5a** (Scheme 4a). We believe that the latter undergoes conjugation-driven transformation to 2,3-dihydropyridine **6a** with the displacement of 1,1-dimethylsilaethylene (highly unstable compound instantly undergoes dimerization to 1,1,3,3-tetramethyl-1,3-disilacyclobutane).^36^ Finally, upon quenching with water, **6a** undergoes protonation with the formation of **2a**·OTf. Since a significant amount of starting material stays unreacted due to the steric reasons (see above), its treatment with water gives DMAP·HOTf, which serves as an acid for the protonation of **6a**. Thus, **6a** is already formed prior to the exposure to air. This hypothesis agrees with our NMR monitoring of the reaction mixture: upon mixing N1-trimethylsilyl-DMAP with *n*-BuLi in THF, one can observe a set of signals fitting the structure **5a** (Figure 7, bottom), which are no longer present after 24 h; instead, characteristic signals of **6a** appear (Figure 7, top).

**Scheme 4:**
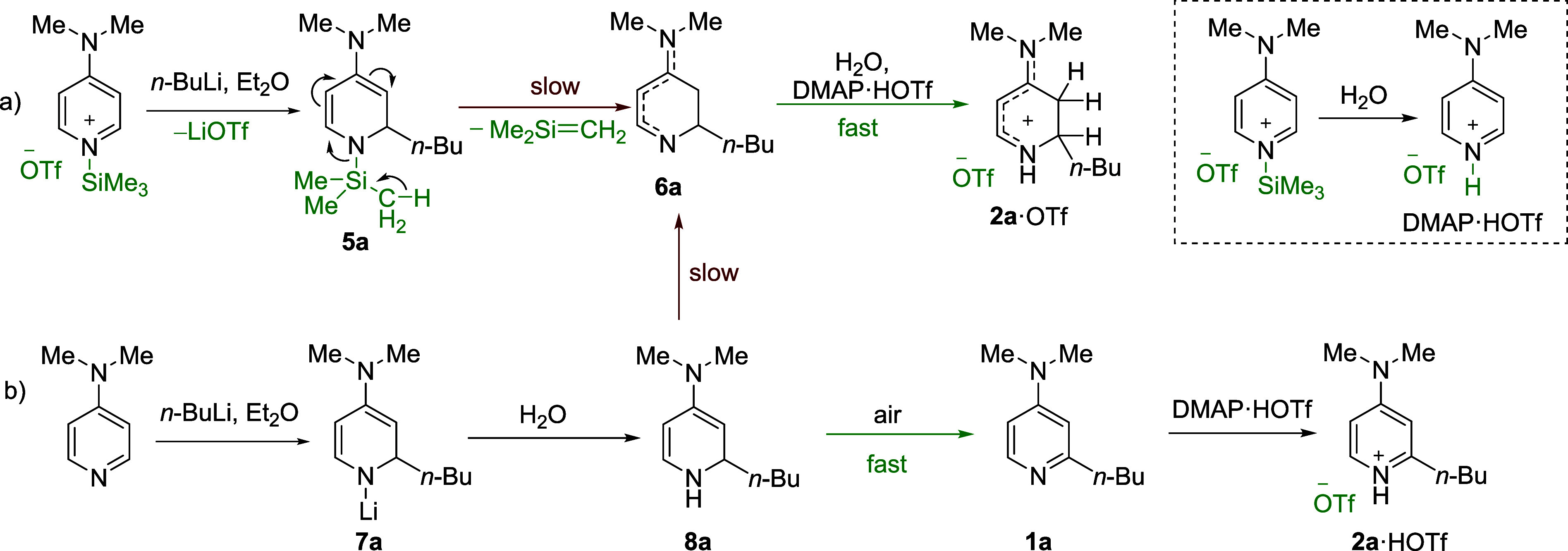
Proposed Mechanisms of the Formation of **2a**·OTf from DMAP and Me_3_Si-DMAP

**Figure 7:**
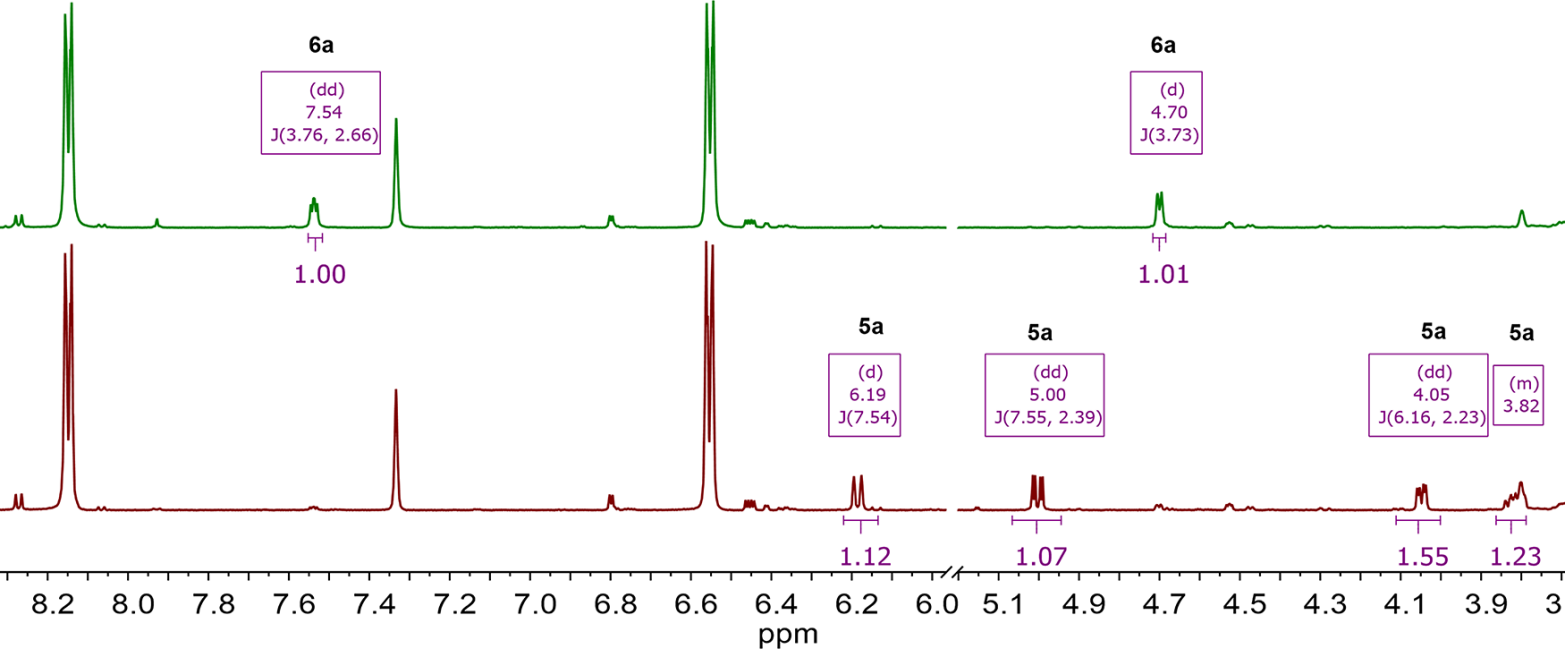
^1^H NMR (THF-*d*_8_, r.t.) monitoring of the reaction between Me_3_Si-DMAP and *n*-BuLi. Bottom spectrum: 30 min after mixing, showing the characteristic upfield-shifted signals of the four dihydropyridine ring protons corresponding to the 1,2-adduct **5a**. Top spectrum: After 24 h, **5a** is converted into the conjugated 2,3-dihydropyridine **6a**, as evidenced by the disappearance of the initial set of signals and the emergence of a new pattern.

In contrast, the reaction of DMAP with *n*-BuLi initially gives adduct **7a**, which has no means of transformation to the 2,3-dihydropyridine structure (Scheme 4b). Only the following treatment with water leads to the formation of 1,2-dihydropyridine **8a**, which slowly isomerizes to 2,3-dihydropyridine **6a**; however, this process is outcompeted by much faster oxidative aromatization into **1a**.

We have also tested 4-methyl- and 4-methoxypyridines in order to confirm the role of NMe_2_ group in the stabilization of 2,3-dihydropyridine backbone. Subsequent treatment with TMSOTf and with *n*-BuLi in diethyl ether led to the formation of aromatic 4-substituted 2-butylpyridines^37^ **7a** and **8a** as main products (Scheme 5). No visible amount of corresponding 2,3-dihydropyridines was detected in the reaction mixture (see Figures S5 and S6 in the Supporting Information). Finally, we have tested 4-pyrrolidinopyridine **9**, 4-piperidinopyridine **10**, and 4-morpholinopyridine **11**. Under the same reaction conditions, they give 2,3-dihydropyridiniums **12**-**14**·OTf (see Figures S7–S9 in the Supporting Information). Based on this, we conclude that the presence of a strongly conjugated vinylogous amidine backbone is related to the abnormal stability of 4-dialkylamino-2,3-dihydropyridines. It should be noted that in contrast to **2**·OTf, **12a**·OTf, and **13a**·OTf, 4-morpholinodihydropyridinium **14a**·OTf undergoes fast transformation to pyridone **3a** upon chromatographic purification. Thus, filtration of the solution of **14a**·OTf through a layer of silica leads to instantaneous transformation to **3a**. On alumina, the reaction proceeds significantly slower allowing isolation of **14a**·OTf with a very low yield.

**Scheme 5:**
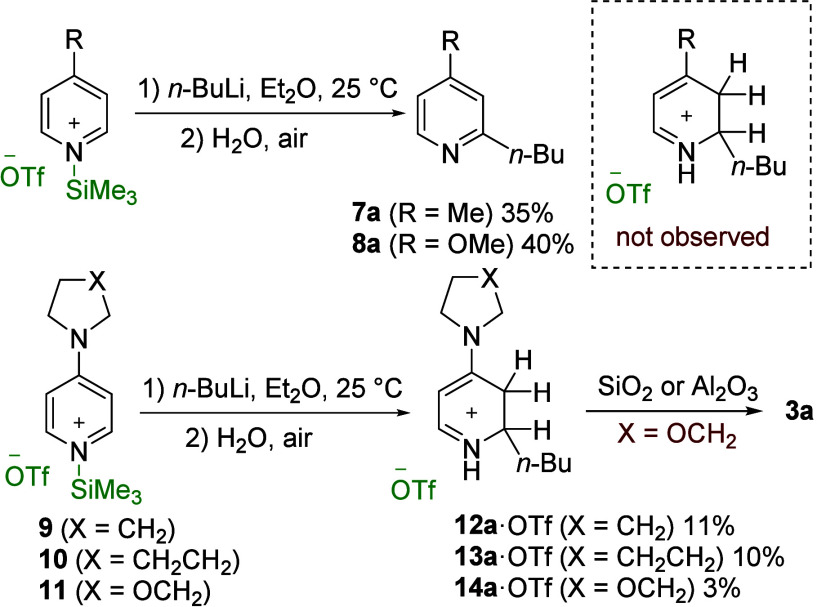
Reactions of N1-Trimethylsilylated 4-Methyl-, 4-Methoxy-, 4-Pyrrolidino-, 4-Piperidino-, and 4-Morpholinopyridines with *n*-BuLi

## Conclusions

In summary, this study unveils a novel and highly stable class of 2,3-dihydro-4-dialkylaminopyridin-1-iums, accessible via N1-trimethylsilylation of 4-dialkylaminopyridines and subsequent reaction with organolithium reagents and establishes N1-silylation combined with a strongly donating C4 substituent as a general strategy to escape the inherent “aromaticity trap” of pyridines. These compounds defy the conventional instability of dihydropyridine intermediates, demonstrating exceptional resistance to rearomatization and deprotonation, which is attributed to effective charge delocalization within the vinylogous amidine backbone of the molecule. The reaction pathway exhibits a critical dependence on the steric profile of the N1-silyl group. Thus, the bulky triisopropylsilyl group effectively blocks nucleophilic addition and unmasks a room-temperature C2(6)-metalation pathway followed by 1,2-silyl migration. Together with ESP analysis, steric accessibility maps, and relative energies of C2 vs C4 adducts, these observations translate into simple design principles: small N1-silyl groups in combination with a strong C4 donor favor access to deeply stabilized nonaromatic states, whereas increased N1-silyl sterics divert reactivity toward metalation-type manifolds. Thus, strategic N1-silylation serves as a powerful tool to break the aromaticity trap, unlocking either stabilized ionic architectures or novel metalation pathways in pyridine chemistry based on steric control.

## Supplementary Material





## Data Availability

The data underlying this study are available in the published article and its Supporting Information.

## Notes

Computational details (electrostatic potentials, potential energy surface scans, accessible surface areas, steric analysis, relative stability of nucleophilic addition products), experimental details, X-ray data and NMR spectra (^1^H and ^13^C­{^1^H}) for all compounds (PDF)
Optimized structures (XYZ)

## †

Artyom A. Yakubenko – Indiana University, Department of Chemistry, 47405 Bloomington, Indiana, United States
