# Supplementary material for: Breaking the Aromaticity Trap: N‑Silylation-Induced Formation of Stable 2,3-Dihydro-4-dialkylaminopyridin-1-iums
Source: J Org Chem. 2026 Jun 25;91(27):9252–60. doi: 10.1021/acs.joc.6c00105 (PMC13366590; doi:10.1021/acs.joc.6c00105)
Supplement: Supplementary file 1 [file jo6c00105_si_001.pdf]

Supporting Information

Breaking the Aromaticity Trap: *N*-Silylation-Induced Formation of Stable 2,3-Dihydro-4-dialkylaminopyridin-1-iums

Valery A. Verkhov,[1] Artyom A. Yakubenko,[1†] Benjamin Begović,[2] Elena Yu. Tupikina,[1] and Alexander S. Antonov*[2]

[1]St. Petersburg State University, Institute of Chemistry, Universitetskii pr. 26, 198504 St. Petersburg, Russian Federation
[2]University of Regensburg, Institute of Organic Chemistry, D-93053 Regensburg, Germany

Table of Contents

Computational details S2
Experimental details S6
X-ray crystallography S15
NMR spectra S18
References S25

Computational details

Computational resources were provided by the Computer Center of Saint-Petersburg University Research Park (http://www.cc.spbu.ru/).The calculations were carried out using the Gaussian16 software package.[1] Geometry optimizations and harmonic vibrational frequencies calculations were performed at the B3LYP/6-311++G(d,p) and PW6B95/def2-TZVPD level of theory.[2–5] Dispersion corrections were incorporated using Grimme's D3 method with Becke-Johnson damping D3(BJ).[6] All structures were checked on the absence of imaginary harmonic vibrational frequencies. Solvent effects were accounted implicitly using the conductor-like polarizable continuum model (CPCM).

The MultiWFN[7] program was used for calculating the surfaces of electron density, electron localization function[8] and molecular electrostatic potential.[9] NBO analysis performed using NBO 7.0 program. The visualization was performed using GaussView. The calculation of the accessible surface area and the volumes of the regions of dispersion attraction was performed using Matlab R2021b.

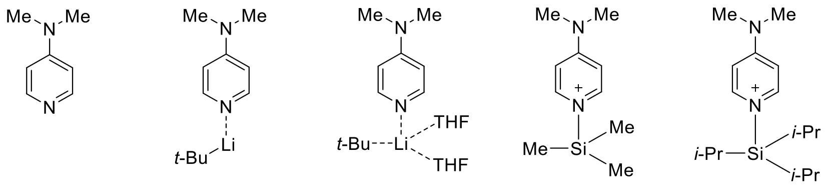

Scheme S1. Systems studied: DMAP is 4-dimethylaminopyridine; *t*-BuLi-DMAP is a 4-dimethylaminopyridine coordinated at the 1-position of *tert*-butyl lithium; *t*-Bu(THF)$_2$Li-DMAP is a 4-dimethylaminopyridine coordinated at the 1-position of *tert*-butyl lithium with 2 tetrahydrofuran molecules; Me$_3$Si-DMAP is a 4-dimethylaminopyridine with an Me$_3$Si substituent at the 1-position; *i*-Pr$_3$Si-DMAP is a 4-dimethylaminopyridine with an *i*-Pr$_3$Si substituent at the 1-position.

Table S1. Computed electrostatic potential (ESP) at pyridine ring carbon atoms. Values are provided for calculations performed in vacuum and using the CPCM(THF) solvation model (kJ/mol). Computational method: PW6B95-D3(BJ)/def2-TZVPD.

Solvent	Position	DMAP	*t*-BuLi-DMAP	*t*-Bu(THF)$_2$Li-DMAP	Me$_3$Si-DMAP	*i*-Pr$_3$Si-DMAP
Vacuum (ε = 1.0)	C2	−57	+19	−12	+338	+322
	C3	−48	+20	−12	+314	+301
	C4	−19	+44	+14	+343	+328
THF (ε = 7.4)	C2	−77	+4	−20	+349	+339
	C3	−54	+7	−13	+322	+316
	C4	−18	+34	+19	+350	+345

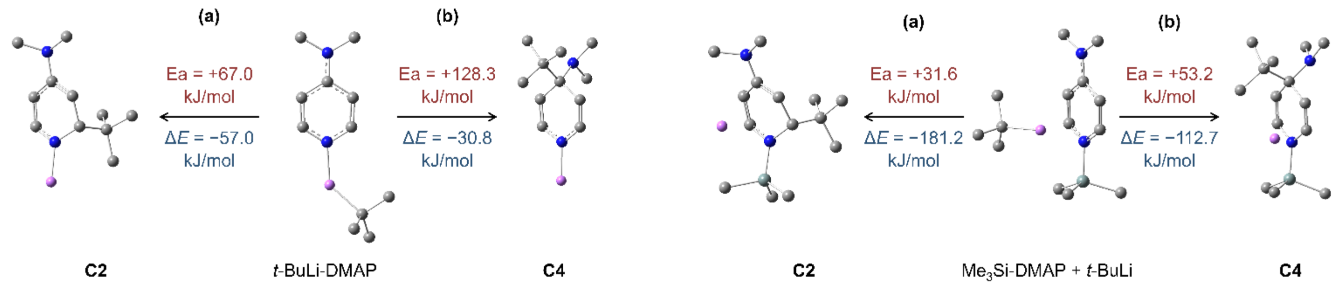

Figure S1. Potential energy surface scans along the (a) C2(DMAP)–C(*t*-Bu) and (b) C4(DMAP)–C(*t*-Bu) reaction coordinates for nucleophilic addition to the C2 and C4 positions of the pyridine ring, respectively. Calculations performed at the B3LYP-D3(BJ)/6-311++G(d,p) level with CPCM(THF) solvation model.

Table S2. Calculated accessible surface areas of pyridine ring carbon atoms. Values represent the surface area of atomic electron density basins defined at the 0.001 a.u. isosurface level. Computational method: PW6B95-D3(BJ)/def2-TZVPD with CPCM(THF) solvation model.

Area	DMAP	*t*-BuLi-DMAP	*t*-Bu$(THF)_2$Li-DMAP	Me_3Si-DMAP	*i*-Pr_3Si-DMAP
S_2, Å^2	16.2	15.9	12.2	14.8	12.5
S_3, Å^2	18.8	18.7	18.7	18.2	18.2
S_4, Å^2	4.6	4.6	4.6	4.4	4.4
S_2 / S_3	0.9	0.8	0.6	0.8	0.7
S_2 / S_4	3.5	3.5	2.7	3.3	2.8

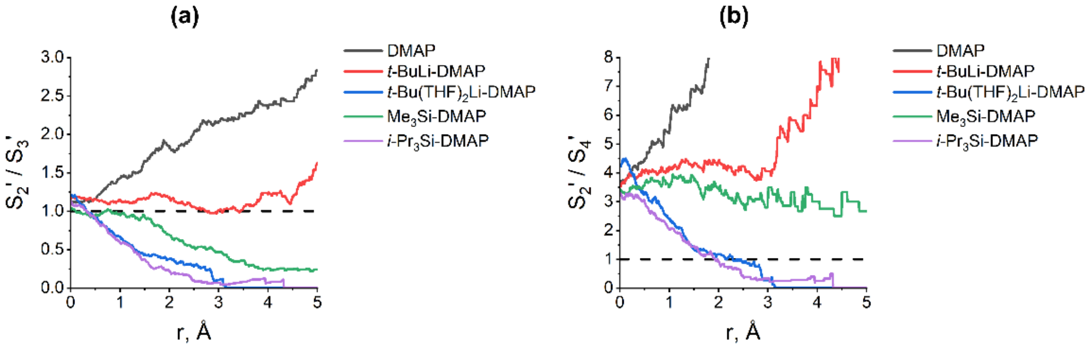

Figure S2. Steric analysis using variable-radius rolling sphere method. Relative accessible surface area ratios of (a) S_2'/S_3' and (b) S_2'/S_4' plotted against sphere radius (r, Å). Computational level: PW6B95-D3(BJ)/def2-TZVPD with CPCM(THF) solvation model.

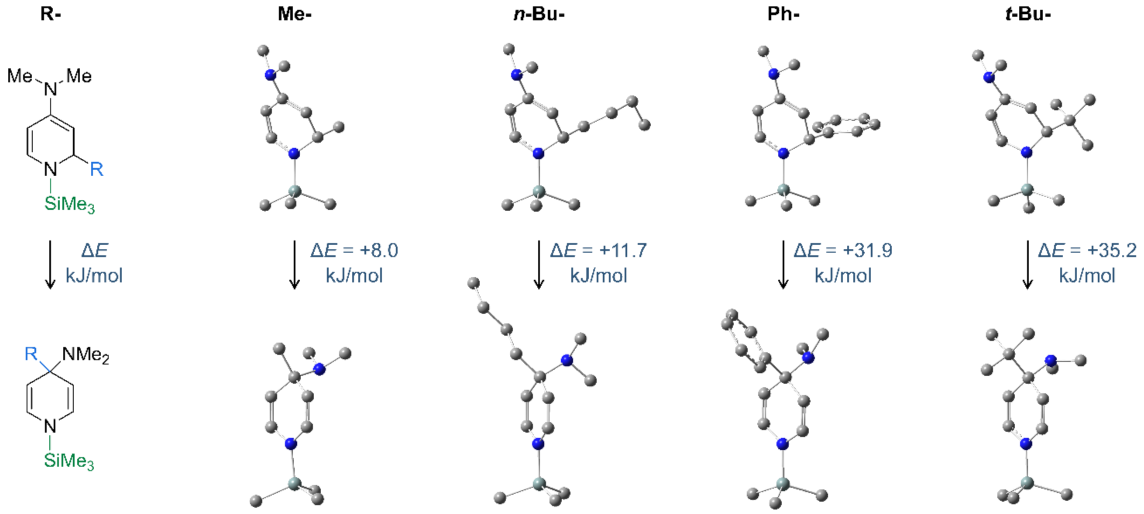

Figure S3. Relative stability of C2 vs C4 nucleophilic addition products for *N*-trimethylsilylated DMAP with different nucleophiles. Top row: C2-addition products; bottom row: C4-addition products. Relative electronic energies (Δ*E*, kJ/mol) between corresponding C2 and C4 adducts are indicated. From left to right: R = Me, *n*-Bu, Ph, *t*-Bu. The increasing steric demand of the nucleophile leads to progressive destabilization of the C4-addition products. Calculations performed at the B3LYP/6-311++G(d,p) level. Hydrogen atoms omitted for clarity.

Experimental details

GENERAL

All solvents used in synthetic procedures (*n*-hexane, benzene, toluene, diethyl ether, and tetrahydrofuran) were dried over sodium/benzophenone under an argon atmosphere and distilled prior to use.

Liquid-state NMR spectra (^{1}H and ^{13}C) were recorded on a Bruker Avance III spectrometer operating at 400 MHz and 100 MHz, respectively, at the Center for Magnetic Resonance of the St. Petersburg State University Research Park and the Center for Chemical Analysis of the Regensburg University. Chemical shifts are reported in ppm relative to tetramethylsilane (TMS).

High-resolution electrospray ionization mass spectrometry was performed on a Bruker maXis spectrometer equipped with an ESI ion source and QTOF analyzer at the Resource Center "Methods for Substance Composition Analysis" of the St. Petersburg State University Research Park and on a Ajilent Q-TOF 6540 UHD spectrometer equipped with an ESI ion source and QTOF analyzer of the Center for Chemical Analysis of the Regensburg University. Measurements were conducted in positive ion mode in an m/z range of 50–1200. Methanol and acetonitrile were used as solvents. The ion source capillary voltage was set to 4000 V, with a nebulizer gas pressure of 1.0 bar and a dry gas flow rate of 4.0 L/min.

Single crystals of **2b**·OTf and **2c**·OTf suitable for X-ray diffraction were grown by slow evaporation from ethyl acetate solutions. Diffraction data were collected on a SuperNova diffractometer (Rigaku Oxford Diffraction) equipped with a HyPix-3000 detector and a micro-focus Cu Kα radiation source (λ = 1.54184 Å) at 100(2) K at the Centre for X-ray Diffraction Studies, St. Petersburg State University Research Park. The structures were solved using SHELXT[10] (Intrinsic Phasing) and refined with SHELXL[11] (Least Squares minimisation) via the Olex2[12] program suite. Crystallographic data have been deposited with the Cambridge Crystallographic Data Centre under deposition numbers 2512080 (**2b**·OTf) and 2512079 (**2c**·OTf).

2-(*n*-butyl)-4-(dimethylamino)-2,3-dihydropyridin-1-ium trifluoromethanesulfonate (2a·OTf) and 2-(*n*-butyl)-2,3-dihydro-4(1*H*)-pyridinone (3a).

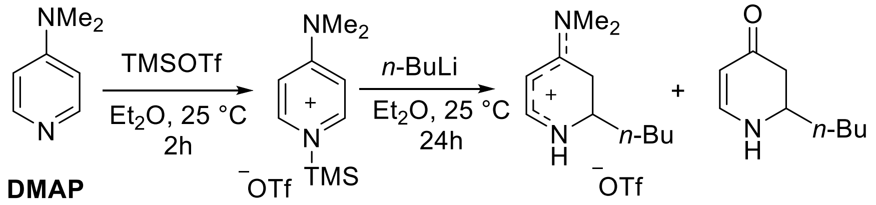

2a·OTf 16% isolated

3a 3% isolated

NMR ratio **DMAP** : **2a**·OTf : **3a** = 1 : 0.3 : 0.1

A flame-dried, argon-flushed 50 mL round-bottom flask equipped with a magnetic stir bar and a silicone septum was charged with 4-dimethylaminopyridine (DMAP, 100 mg, 0.819 mmol) and dry diethyl ether (20 mL). Me_3SiOTf (158 μL, 0.819 mmol) was added via syringe. The mixture was stirred at room temperature for 2 h to afford white precipitate of Me_3Si-$DMAP^{+}OTf^{-}$. 1.6 M solution of *n*-butyllithium in hexanes (0.51 mL, 0.819 mmol) was added dropwise via syringe at 25 °C. The mixture was stirred at the same temperature for 24 h and quenched by the addition of water in air. Organic layer was separated and aqueous layer was extracted with dichloromethane (2 × 20 mL). The combined organic layers were dried over anhydrous Na_2SO_4, and the solvent was evaporated to dryness. The residue was purified by

TLC with Al_2O_3 and tetrahydrofuran as eluent to give **2a**·OTf as yellow crystals (44 mg, 16%, R_f = 0–0.06) and **3a** as yellow crystals (3.2 mg, 3%, R_f = 0.06–0.12).

2-(*n*-butyl)-4-(dimethylamino)-2,3-dihydropyridin-1-ium trifluoromethanesulfonate (**2a**·OTf):

^{1}H NMR (400 MHz, $CDCl_3$): δ = 8.87 (s, 1 H), 7.65 (m, 1 H), 5.14 (d, ^3J = 6.6 Hz, 1 H), 3.74 (m, 1 H), 3.32 (s, 3 H), 3.23 (s, 3 H), 2.89 (dd, ^2J = 16.9, ^3J = 6.3 Hz 1 H), 2.58 (dd, ^2J = 16.9, ^3J = 12.7 Hz 1 H), 1.81 (m, 1 H), 1.68 (m, 1 H), 1.40 (m, 4 H), 0.93 (t, ^3J = 6.9 Hz, 3 H) ppm.

^{13}C{^{1}H} NMR (100 MHz, $CDCl_3$): δ = 165.9, 156.6, 120.6 (q, J_{CF} = 320.2 Hz) 87.8, 50.4, 41.2, 40.7, 32.3, 30.8, 27.2, 22.4, 13.8 ppm.

HRMS (ESI) m/z: $[M-CF_3SO_3]^+$ Calcd for $C_{11}H_{21}N_2^+$: 181.1699; Found: 181.1692.

Mp 117 – 118 °C.

2-(*n*-butyl)-2,3-dihydro-4(1*H*)-pyridinone (**3a**):

Spectral data is in agreement with previously published.[13]

^{1}H NMR (400 MHz, $CDCl_3$): δ = 7.19 (m, 1 H), 5.66 (s, 1 H), 4.97 (d, ^3J = 7.3 Hz, 1 H), 3.64 (m, 1 H), 2.37 (m, 2 H), 1.62 (m, 2 H), 1.34 (m, 4 H), 0.91 (m, 3 H) ppm.

2-(*sec*-butyl)-4-(dimethylamino)-2,3-dihydropyridin-1-ium trifluoromethanesulfonate (2b·OTf).

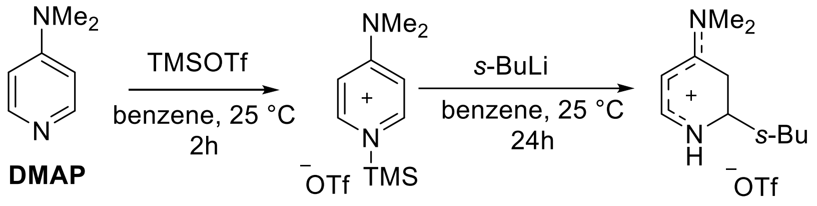

2b·OTf
8% isolated

NMR ratio **DMAP** : **2b**·OTf = 1 : 0.4

A flame-dried, argon-flushed 50 mL round-bottom flask equipped with a magnetic stir bar and a silicone septum was charged with 4-dimethylaminopyridine (DMAP, 100 mg, 0.819 mmol) and dry benzene (20 mL). Me_3SiOTf (158 μL, 0.819 mmol) was added via syringe. The mixture was stirred at room temperature for 2 h to afford white precipitate of $Me_3Si\text{-}DMAP^+OTf^-$. 1.4 M solution of *sec*-butyllithium in cyclohexane (0.59 mL, 0.819 mmol) was added dropwise via syringe at 25 °C. The mixture was stirred at the same temperature for 24 h and quenched by the addition of water in air. Organic layer was separated and aqueous layer was extracted with dichloromethane (2 × 20 mL). The combined organic layers were dried over anhydrous Na_2SO_4, and the solvent was evaporated to dryness. The residue was purified by TLC with Al_2O_3 and tetrahydrofuran as eluent to give **2b**·OTf as yellow crystals (22 mg, 8%). Rf = 0 – 0.06 (Al_2O_3, THF). Mp 70 – 71°C.

^{1}H NMR (400 MHz, $CDCl_3$): δ = 8.50 (s, 1 H), 7.56 (m, 1 H), 5.07 (dd, ^3J = 6.7 Hz, ^4J = 1.7 Hz, 1 H), 3.59 (m, 1 H), 3.25 (s, 3 H), 3.16 (s, 3 H), 2.71 (m, 1 H), 2.56 (m, 1 H), 1.77 (m, 1 H), 1.45 (m, 1 H), 1.23 (m, 1 H), 0.90 (m, 6 H) ppm.

^{13}C{^{1}H} NMR (100 MHz, $CDCl_3$): δ = 166.5, 156.8, 120.6 (q, J_{CF} = 320.1 Hz), 87.8, 54.6, 41.2, 40.7, 36.8, 36.5, 28.3, 25.1, 14.4 ppm.

HRMS (ESI) m/z: $[M-CF_3SO_3]^+$ Calcd for $C_{11}H_{21}N_2^+$: 181.1699; Found: 181.1684

2-(*tert*-butyl)-4-(dimethylamino)-2,3-dihydropyridin-1-ium trifluoromethanesulfonate (2c·OTf).

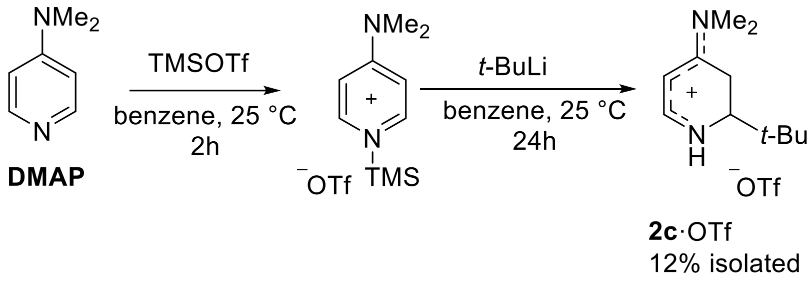

NMR ratio **DMAP** : **2b**·OTf = 1 : 0.5

A flame-dried, argon-flushed 50 mL round-bottom flask equipped with a magnetic stir bar and a silicone septum was charged with 4-dimethylaminopyridine (DMAP, 100 mg, 0.819 mmol) and dry benzene (20 mL). Me_3SiOTf (158 µL, 0.819 mmol) was added via syringe. The mixture was stirred at room temperature for 2 h to afford white precipitate of Me_3Si-$DMAP^+OTf^-$. 1.7 M solution of *tert*-butyllithium in pentane (0.48 mL, 0.819 mmol) was added dropwise via syringe at 25 °C. The mixture was stirred at the same temperature for 24 h and quenched by the addition of water in air. Organic layer was separated and aqueous layer was extracted with dichloromethane (2 × 20 mL). The combined organic layers were dried over anhydrous Na_2SO_4, and the solvent was evaporated to dryness. The residue was purified by TLC on Al_2O_3 with tetrahydrofuran as eluent to give **2c**·OTf as yellow crystals (32 mg, 12%). Rf = 0–0.06 (Al_2O_3, THF). Mp 82 – 83 °C.

^{1}H NMR (400 MHz, $CDCl_3$): δ = 8.43 (s, 1 H), 7.62 (dd, ^3J = 6.7 Hz, ^4J = 1.3 Hz, 1 H), 5.07 (d, ^3J = 6.7 Hz, 1 H), 3.33 (m, 1 H), 3.25 (s, 3 H), 3.15 (s, 3 H), 2.76 (dd, ^2J = 16.9 Hz, ^3J = 6.2 Hz, 1 H), 2.52 (m, 1 H), 0.97 (s, 9 H) ppm.

^{13}C{^{1}H} NMR (100 MHz, $CDCl_3$): δ = 166.5, 157.0, 120.6 (q, J_{CF} = 320.2 Hz), 87.7, 59.4, 41.3, 40.6, 33.3, 25.8 ppm.

HRMS (ESI) m/z: $[M-CF_3SO_3]^+$ Calcd for $C_{11}H_{21}N_2^+$: 181.1699; Found: 181.1686

2-(*n*-butyl)-4-(pyrrolidino)-2,3-dihydropyridin-1-ium trifluoromethanesulfonate (12a·OTf).

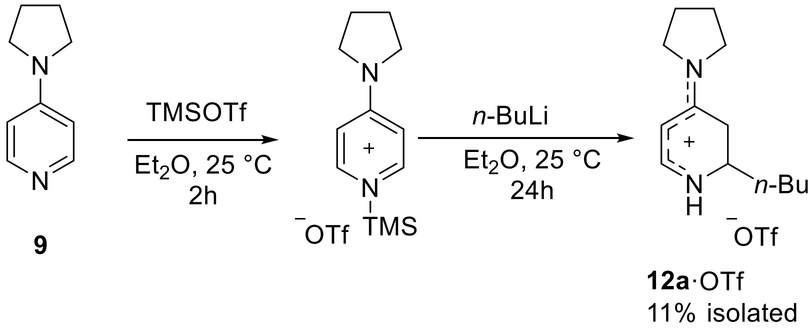

NMR ratio **9** : **12a**·OTf = 1 : 0.9

A flame-dried, argon-flushed 50 mL round-bottom flask equipped with a magnetic stir bar and a silicone septum was charged with 4-pyrrolidinopyridine (100 mg, 0.676 mmol) and dry diethyl ether (20 mL). Me_3SiOTf (130 µL, 0.676 mmol) was added via syringe. The mixture was stirred at room temperature for 2 h to afford white precipitate of Me_3Si-(4-pyrrolidinopyridine)$^+OTf^-$. 1.6 M solution of *n*-butyllithium in hexanes (0.42 mL, 0.676 mmol) was added dropwise via syringe at 25 °C. The mixture was stirred at the same temperature for 24 h and quenched by the addition of water in air. Organic layer was separated and aqueous layer was extracted with dichloromethane (2 × 20 mL). The combined organic layers were dried over anhydrous Na_2SO_4, and the solvent was evaporated to dryness. The residue was filtered through the layer of silica using MeCN as solvent. The solvent was evaporated to dryness, and the residue was

purified by TLC with Al_2O_3 and tetrahydrofuran as eluent to **12a**·OTf as yellow crystals (27 mg, 11%, R_f = 0.1). Mp 59–61 °C.

^{1}H NMR (400 MHz, $CDCl_3$): δ = 8.71 (s, 1H), 7.53 (t, ^3J = 7.0 Hz, 1H), 4.96 (d, ^3J = 6.6 Hz, 1H), 3.74 – 3.37 (m, 5H), 2.79 (dd, ^3J = 17.0 Hz, ^4J = 6.2 Hz, 1H), 2.52 (dd, ^3J = 17.1, 12.9, 1H), 2.12 – 1.96 (m, 4H), 1.80 – 1.68 (m, 1H), 1.65 – 1.51 (m, 1H), 1.36 – 1.25 (m, 4H), 0.87 – 0.82 (m, 3H).

$^{13}C\{^1H\}$ NMR (100 MHz, $CDCl_3$): δ = 163.12, 156.34, 125.63, 120.7 (q, J_{CF} = 320.0 Hz), 88.37, 50.47, 50.10, 49.85, 32.49, 32.14, 27.34, 25.01, 24.60, 22.51, 13.91.

HRMS (ESI) m/z: $[M{-}CF_3SO_3]^+$ Calcd for $C_{13}H_{23}N_2^+$: 207.1861; Found: 207.1862

2-(*n*-butyl)-4-(piperidino)-2,3-dihydropyridin-1-ium trifluoromethanesulfonate (13a·OTf).

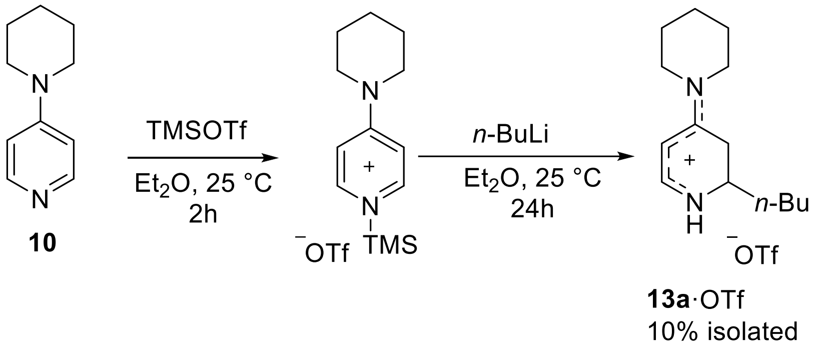

NMR ratio **10** : **13a**·OTf = 1 : 0.6

A flame-dried, argon-flushed 50 mL round-bottom flask equipped with a magnetic stir bar and a silicone septum was charged with 4-piperidinopyridine (100 mg, 0.617 mmol) and dry diethyl ether (20 mL). Me_3SiOTf (119 µL, 0.617 mmol) was added via syringe. The mixture was stirred at room temperature for 2 h to afford white precipitate of Me_3Si-(4-piperidinopyridine)$^+$OTf$^-$. 1.6 M solution of *n*-butyllithium in hexanes (0.39 mL, 0.617 mmol) was added dropwise via syringe at 25 °C. The mixture was stirred at the same temperature for 24 h and quenched by the addition of water in air. Organic layer was separated and aqueous layer was extracted with dichloromethane (2 × 20 mL). The combined organic layers were dried over anhydrous Na_2SO_4, and the solvent was evaporated to dryness. The residue was filtered through the layer of silica using MeCN as solvent. The solvent was evaporated to dryness, and the residue was purified by TLC with Al_2O_3 and tetrahydrofuran as eluent to give **13a**·OTf as dark yellow wax (23 mg, 10%, R_f = 0.1).

^{1}H NMR (400 MHz, $CDCl_3$): δ = 8.94 (s, 1H), 7.65 (t, ^3J = 7.1 Hz, 1H), 5.21 (d, ^3J = 6.8 Hz, 1H), 3.87 – 3.49 (m, 5H), 2.83 (dd, ^3J =16.6 Hz, ^4J = 6.3 Hz, 1H), 2.53 (dd, ^3J = 16.7 Hz, ^4J =12.2 Hz, 1H), 1.87 – 1.59 (m, 8H), 1.42 – 1.27 (m, 4H), 0.91 (t, ^3J = 7.1 Hz, 3H).

$^{13}C\{^1H\}$ NMR (100 MHz, $CDCl_3$): δ = 164.11, 157.15, 125.59 – 115.60 (m), 87.83, 50.89, 49.92, 49.63, 32.74, 30.95, 27.64, 27.03, 25.93, 24.08, 22.83, 14.23.

HRMS (ESI) m/z: m/z $[M{-}CF_3SO_3]^+$ Calcd for $C_{14}H_{25}N_2^+$: 221.2018; Found: 221.2018

2-(*n*-butyl)-4-(morpholino)-2,3-dihydropyridin-1-ium trifluoromethanesulfonate (14a·OTf).

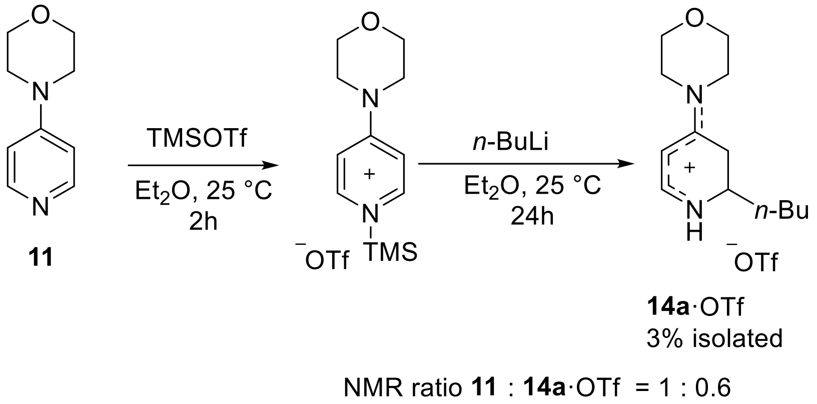

A flame-dried, argon-flushed 50 mL round-bottom flask equipped with a magnetic stir bar and a silicone septum was charged with 4-morpholinopyridine (100 mg, 0.610 mmol) and dry diethyl ether (20 mL). Me_3SiOTf (118 μL, 0.610 mmol) was added via syringe. The mixture was stirred at room temperature for 2 h to afford white precipitate of Me_3Si-(4-morpholinopyridine)$^+$OTf$^-$. 1.6 M solution of *n*-butyllithium in hexanes (0.38 mL, 0.610 mmol) was added dropwise via syringe at 25 °C. The mixture was stirred at the same temperature for 24 h and quenched by the addition of water in air. Organic layer was separated and aqueous layer was extracted with dichloromethane (2 × 20 mL). The combined organic layers were dried over anhydrous Na_2SO_4, and the solvent was evaporated to dryness. The residue was purified by TLC with Al_2O_3 and tetrahydrofuran as eluent to give **14a**·OTf as yellow oil (7 mg, 3%, R_f = 0).

^{1}H NMR (400 MHz, $CDCl_3$): δ = 8.86 (s, 1H), 7.60 (t, ^3J = 6.8 Hz, 1H), 5.24 (d, ^3J = 6.7 Hz, 1H), 3.84 – 3.50 (m, 9H), 2.86 (dd, ^3J = 16.9 Hz, ^4J = 6.4, 1H), 2.52 (dd, ^3J = 16.9 Hz, ^4J = 12.7, 1H), 1.80 – 1.67 (m, 1H), 1.66 – 1.55 (m, 1H), 1.38 – 1.21 (m, 4H), 0.84 (t, ^3J = 7.1 Hz, 3H).

$^{13}C\{^1H\}$ NMR (100 MHz, $CDCl_3$): δ = 164.83, 157.43, 125.52 – 115.48 (m), 87.95, 66.46, 65.83, 50.67, 48.10, 47.93, 32.38, 30.42, 27.18, 22.46, 13.86.

HRMS (ESI) m/z: m/z [M−CF_3SO_3]$^+$ Calcd for $C_{13}H_{23}N_2O^+$: 223.1805; Found: 223.1813

Reaction of 1-triisopropylsilyl-4-dimethylaminopyridinium triflate with alkyllithiums

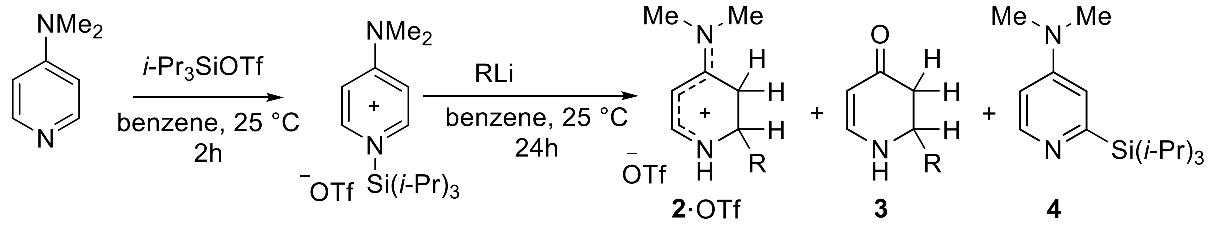

A flame-dried, argon-flushed 50 mL round-bottom flask equipped with a magnetic stir bar and a silicone septum was charged with 4-dimethylaminopyridine (DMAP, 100 mg, 0.819 mmol) and dry benzene (20 mL). *i*-Pr_3SiOTf (228 μL, 0.819 mmol) was added via syringe. The mixture was stirred at room temperature for 2 h to afford white precipitate of *i*-Pr_3Si-DMAP$^+$OTf$^-$. 1.6 M solution of *n*-butyllithium in hexanes (0.51 mL, 0.819 mmol) or 1.4 M solution of *sec*-butyllithium in cyclohexane (0.59 mL, 0.819 mmol) or 1.7 M solution of *tert*-butyllithium in pentane (0.48 mL, 0.819 mmol) was added dropwise via syringe at 25 °C. The mixture was stirred at the same temperature for 24 h and quenched by the addition of water in air. Organic layer was separated and aqueous layer was extracted with dichloromethane (2 × 20 mL). The combined organic layers were dried over anhydrous Na_2SO_4, and the solvent was evaporated to dryness. The residue was subjected to NMR measurements, see Table 3 in manuscript.

Reaction of 1-trimethylsilyl-4-methylpyridinium triflate with *n*-butyllithium

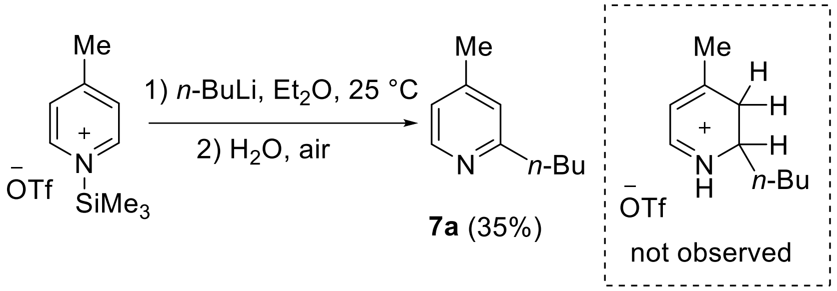

A flame-dried, argon-flushed 50 mL round-bottom flask equipped with a magnetic stir bar and a silicone septum was charged with 4-methylpyridine (100 mg, 1.07 mmol) and anhydrous Et_2O (20 mL). Me_3SiOTf (207 µL, 0.92 mmol) was added via syringe (use Hamilton syringe). The mixture was stirred at room temperature for 2 h. 1.6 M solution of *n*-butyllithium in hexanes (0.67 mL, 1.07 mmol) was added dropwise via syringe. The reaction was stirred at room temperature for 24 h. The reaction was quenched by the addition of water in air. The mixture was extracted with dichloromethane (2 × 20 mL). The combined organic layers were dried over anhydrous Na_2SO_4, after which the solvent is evaporated to dryness. The residue was subjected to NMR measurements (Figure S5).

Reaction of 1-trimethylsilyl-4-methoxypyridinium triflate with *n*-butyllithium

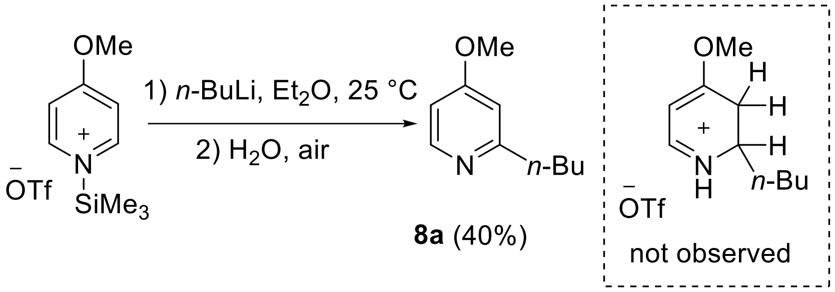

A flame-dried, argon-flushed 50 mL round-bottom flask equipped with a magnetic stir bar and a silicone septum was charged with 4-methoxypyridine (100 mg, 0.92 mmol) and anhydrous Et_2O (20 mL). Me_3SiOTf (177 µL, 0.92 mmol) was added via syringe (use Hamilton syringe). The mixture was stirred at room temperature for 2 h. 1.6 M solution of *n*-butyllithium in hexanes (0.52 mL, 0.92 mmol) was added dropwise via syringe. The reaction was stirred at room temperature for 24 h. The reaction was quenched by the addition of water in air. The mixture was extracted with dichloromethane (2 × 20 mL). The combined organic layers were dried over anhydrous Na_2SO_4, after which the solvent is evaporated to dryness. The residue was subjected to NMR measurements (Figure S6).

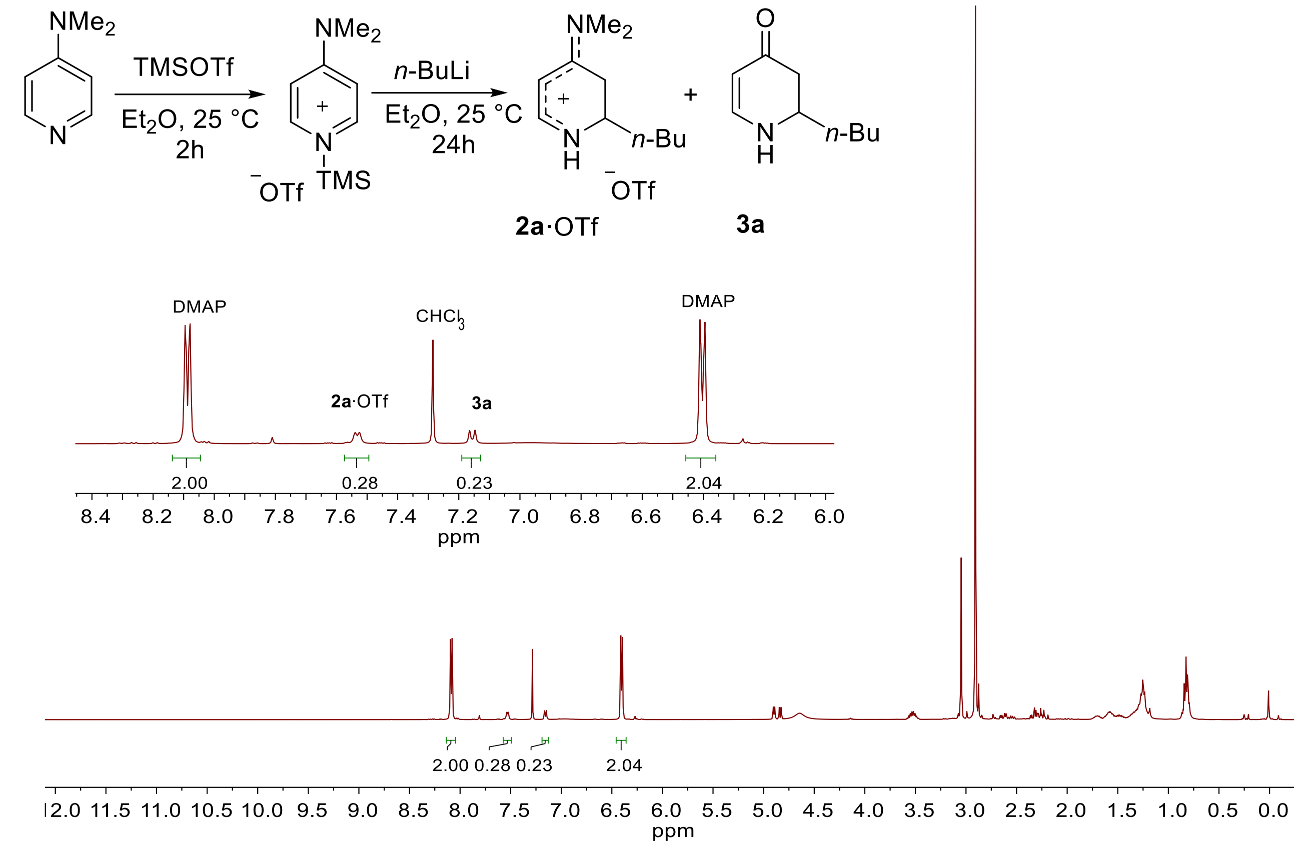

Figure S4. Typical ^{1}H NMR spectrum of the mixture obtained after the reaction of Me_3Si-DMAP^{+}OTf^{-} with organolithiums (400 MHz, $CDCl_3$)

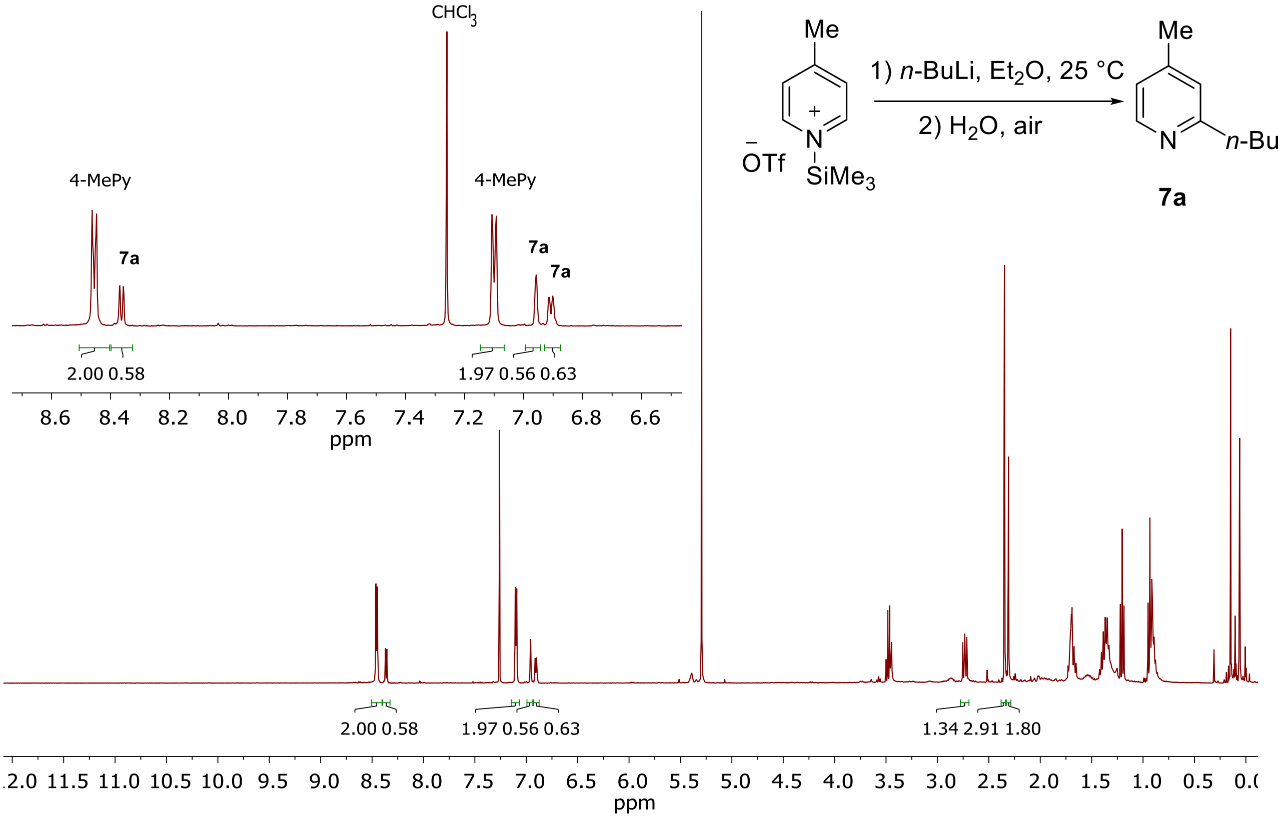

Figure S5. Typical ^{1}H NMR spectrum of the mixture obtained after the reaction of Me_3Si-(4-methylpyridine)$^{+}$OTf^{-} with *n*-BuLi (400 MHz, $CDCl_3$)

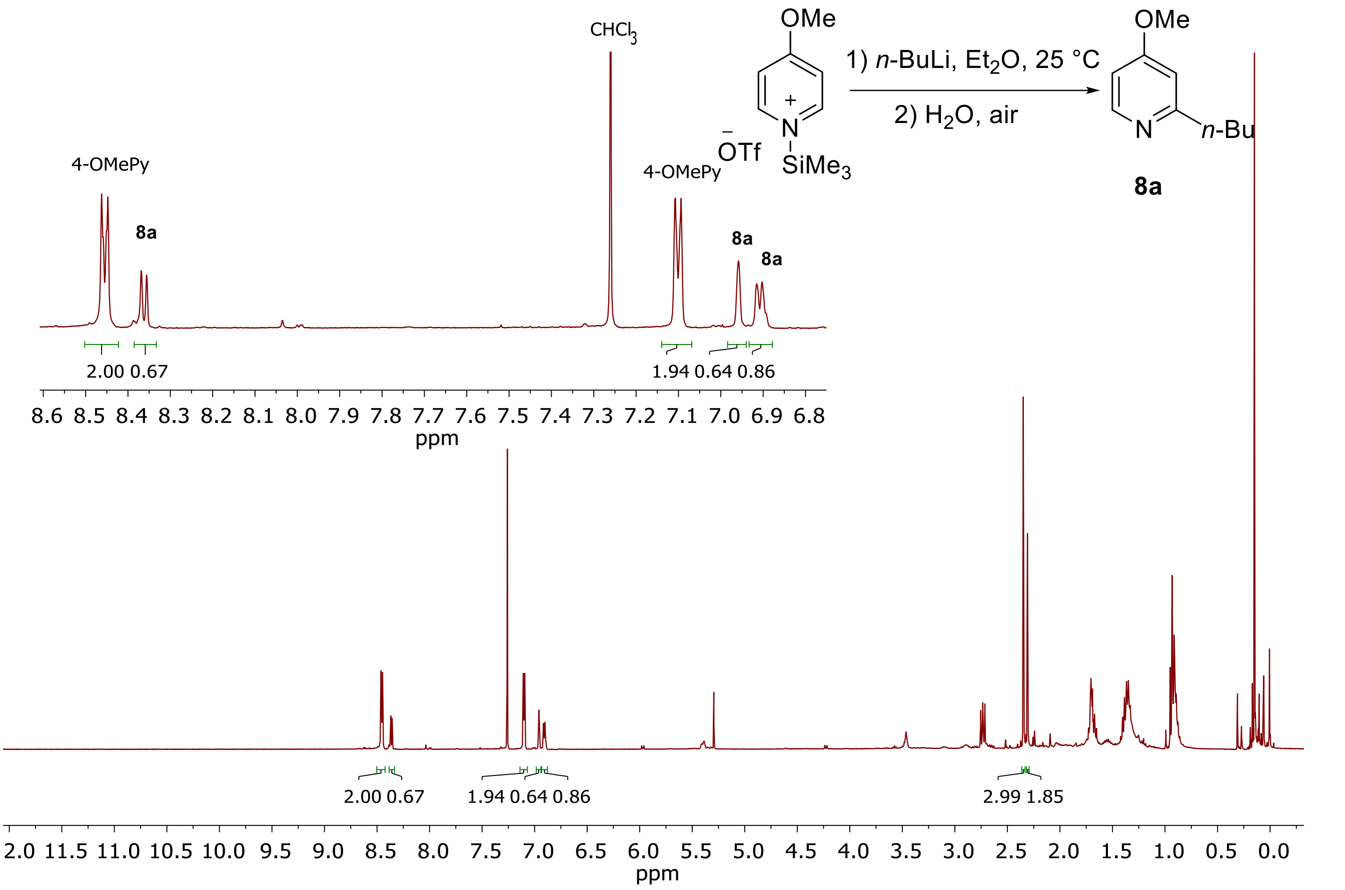

Figure S6. Typical ^{1}H NMR spectrum of the mixture obtained after the reaction of Me_3Si-(4-methoxypyridine)$^+$OTf$^-$ with *n*-BuLi (400 MHz, $CDCl_3$)

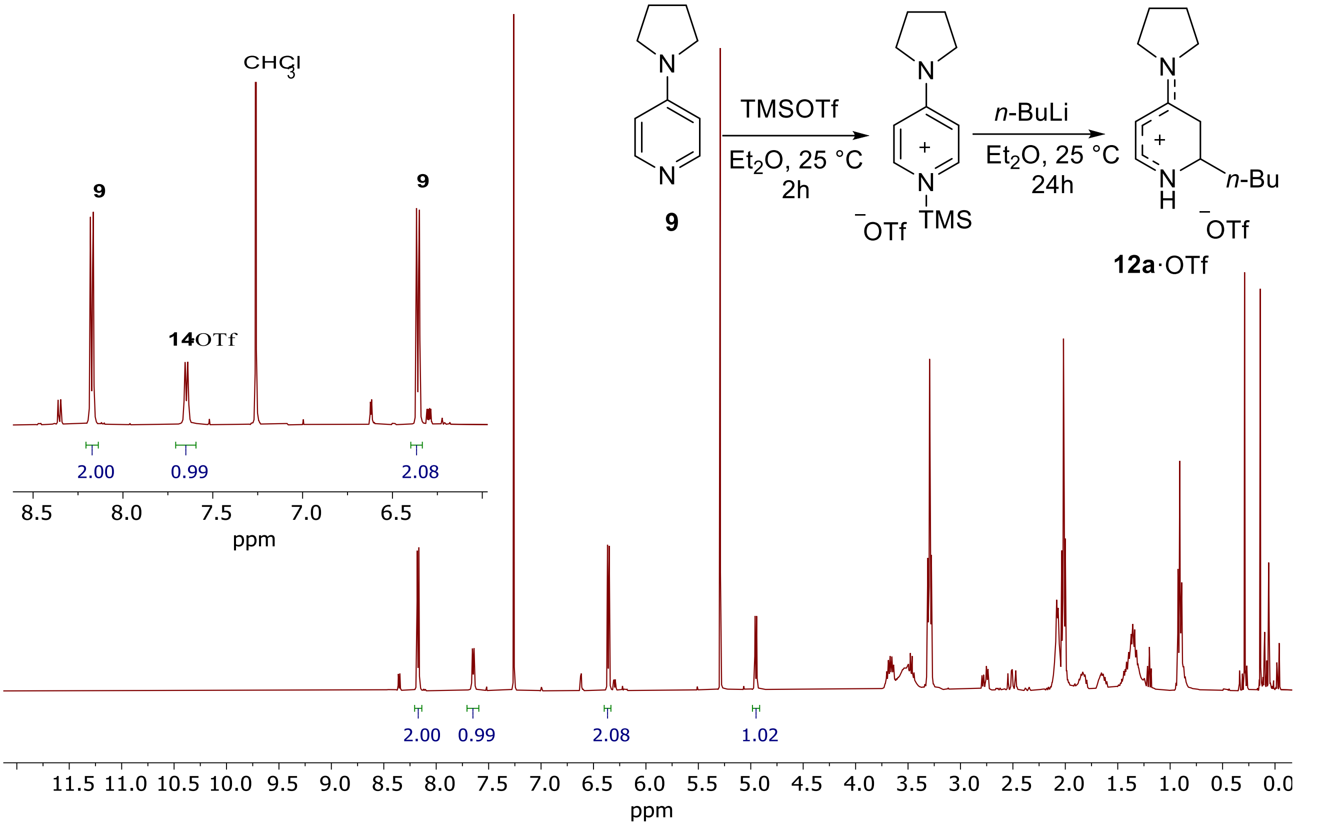

Figure S7. Typical ^{1}H NMR spectrum of the mixture obtained after the reaction of Me_3Si-(4-pyrrolidinopyridine)$^+$OTf$^-$ with *n*-BuLi (400 MHz, $CDCl_3$)

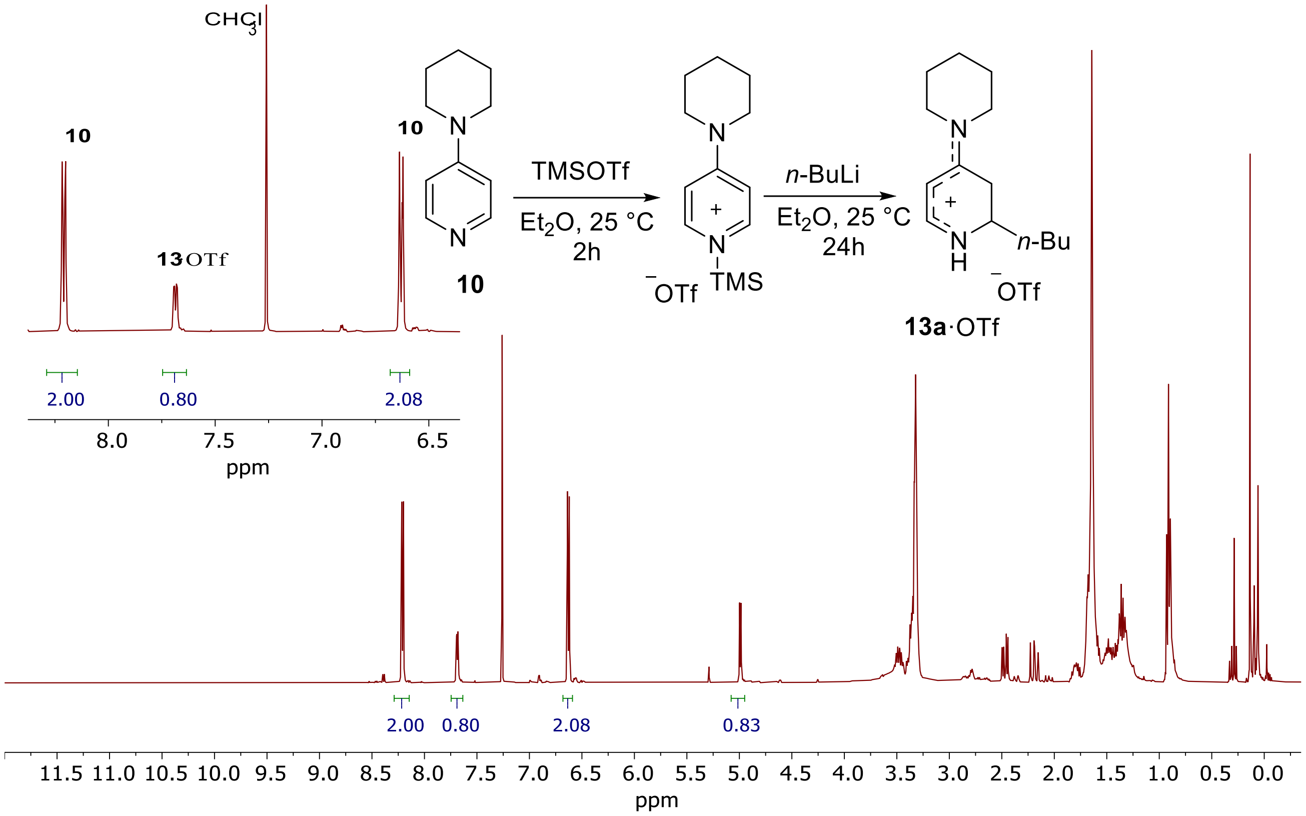

Figure S8. Typical ^{1}H NMR spectrum of the mixture obtained after the reaction of Me_3Si-(4-piperidino pyridine)$^+OTf^-$ with *n*-BuLi (400 MHz, $CDCl_3$)

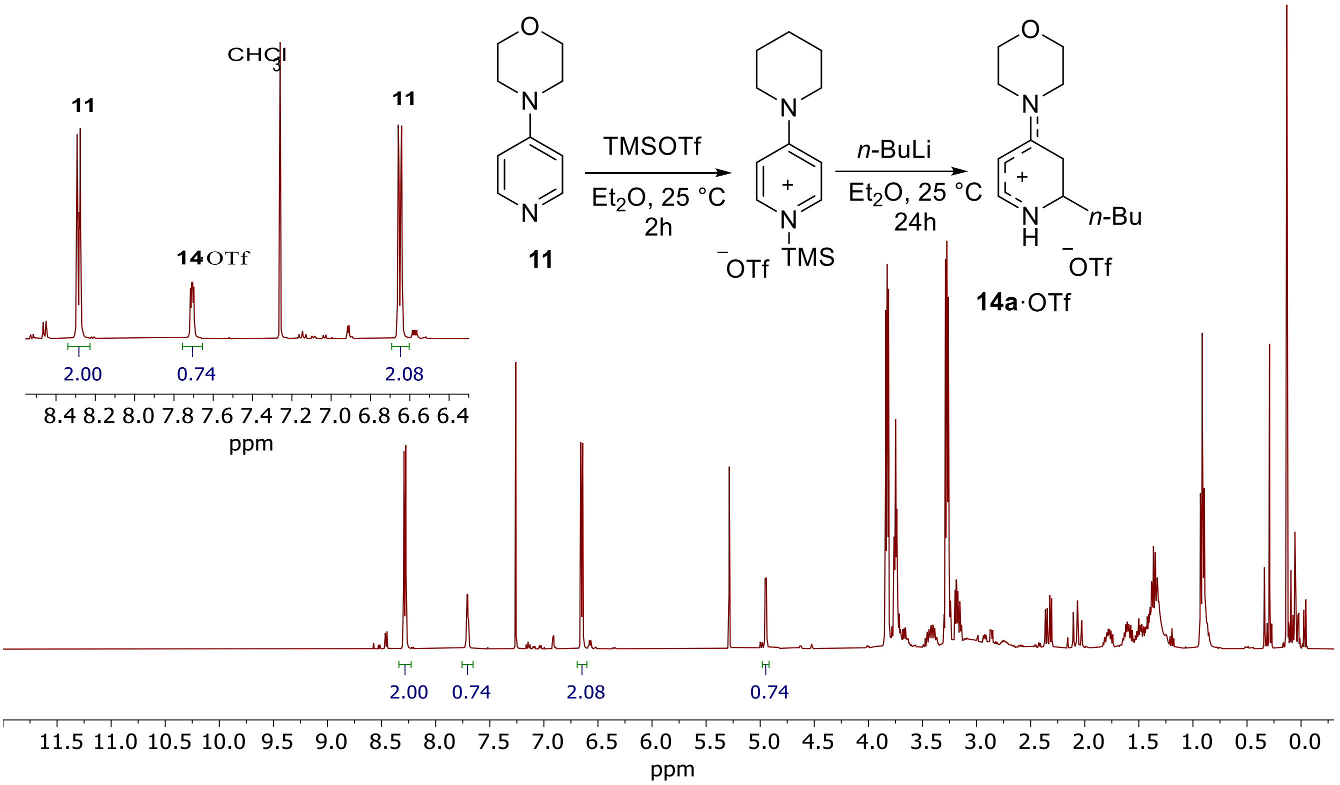

Figure S9. Typical ^{1}H NMR spectrum of the mixture obtained after the reaction of Me_3Si-(4-morpholinopyridine)$^+OTf^-$ with *n*-BuLi (400 MHz, $CDCl_3$)

Table S3. Reactions of Me_3Si-DMAP with organolithium reagents.

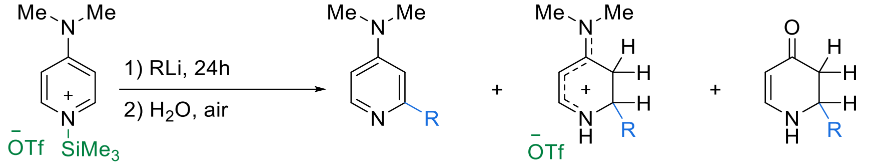

Run	Conditions	R	Product ratio			
			**1**	**2**	**3**	**DMAP**
1	1 eq. *n*-BuLi, *n*-hexane, –25 °C	*n*-Bu	–	–	–	1
2	1 eq. *n*-BuLi, *n*-hexane, +25 °C	*n*-Bu	–	–	–	1
3	1 eq. *n*-BuLi, toluene, –25 °C	*n*-Bu	–	0.08	0.01	1
4	1 eq. *n*-BuLi, benzene, +25 °C	*n*-Bu	–	0.09	–	1
5	1 eq. *n*-BuLi, ether, –25 °C	*n*-Bu	–	0.26	0.07	1
6	1 eq. *n*-BuLi, ether, +25 °C	*n*-Bu	–	0.30	0.10	1
7	1 eq. *n*-BuLi, THF, –25 °C	*n*-Bu	–	0.14	0.01	1
8	1 eq. *n*-BuLi, THF, +25 °C	*n*-Bu	–	0.11	–	1
9	1 eq. *n*-BuLi, 1.0 eq. TMEDA, *n*-hexane, –25 °C	*n*-Bu	–	0.11	–	1
10	1 eq. *n*-BuLi, 1.0 eq. TMEDA, benzene, +25 °C	*n*-Bu	–	0.32	0.09	1
11	1 eq. *n*-BuLi, 1.0 eq. PMDTA, benzene, +25 °C	*n*-Bu	–	0.06	–	1
12	1 eq. *n*-BuLi, 0.2 eq. PMDTA, benzene, +25 °C	*n*-Bu	–	0.19	0.03	1
13	1 eq. *n*-BuLi, 1.0 eq. TMEDA, ether, +25 °C	*n*-Bu	–	0.13	–	1
14	2 eq. *n*-BuLi, benzene, +25 °C	*n*-Bu	0.15	0.13	0.04	1
15	2 eq. *n*-BuLi, 1.0 eq. TMEDA, benzene, +25 °C	*n*-Bu	0.95	0.75	0.15	1
16	2 eq. *n*-BuLi, ether, –25 °C	*n*-Bu	2.14	1.08	1.14	1
17	2 eq. *n*-BuLi, ether, +25 °C	*n*-Bu	1.11	0.60	0.48	1
18	2 eq. *n*-BuLi, 1.0 eq. TMEDA, ether, +25 °C	*n*-Bu	0.63	0.73	0.22	1
19	1 eq. *s*-BuLi, benzene, +25 °C	*s*-Bu	–	0.35	–	1
20	1 eq. *s*-BuLi, 0.2 eq. PMDTA, benzene, +25 °C	*s*-Bu	–	0.21	–	1
21	1 eq. *s*-BuLi, ether, +25 °C	*s*-Bu	–	0.30	–	1
22	1 eq. *t*-BuLi, benzene, +25 °C	*t*-Bu	–	0.54	–	1
23	1 eq. *t*-BuLi, 0.2 eq. PMDTA, benzene, +25 °C	*t*-Bu	–	0.30	–	1
24	1 eq. *t*-BuLi, ether, +25 °C	*t*-Bu	–	0.20	–	1
25	1 eq. *t*-BuLi, THF, –25 °C	*t*-Bu	–	0.26	–	1
26	1 eq. EtLi, ether, +25 °C	Et	–	<0.0	–	1
27	1 eq. Me_3SiCH_2Li, ether, +25 °C	Me_3SiCH_2	–	<0.0	–	1
28	1 eq. MeLi, ether, +25 °C	Me	–	–	–	1
29	1 eq. MeLi, 0.2 eq. PMDTA, ether, +25 °C	Me	–	–	–	1
30	1 eq. MeLi, THF, –25 °C	Me	–	–	–	1
31	1 eq. MeLi, THF, +25 °C	Me	–	–	–	1
32	1 eq. MeLi, THF, +50 °C	Me	–	–	0.03	1
33	1 eq. PhLi, ether, +25 °C	Ph	–	–	–	1
34	1 eq. PhLi, 0.2 eq. PMDTA, ether, +25 °C	Ph	–	–	–	1
36	1 eq. PhLi, THF, +50 °C	Ph	–	–	0.01	1

X-RAY CRYSTALLOGRAPHY

Table S4. Crystallographic data and structure refinement for **2b·OTf** and **2c·OTf**

Compound	**2b·OTf**	**2c·OTf**
Empirical formula	$C_{12}H_{21}N_2O_3F_3S$	$C_{14}H_{27}F_3N_2O_4S_2$
Formula weight	330.37	408.49
Temperature/K	99.9(9)	100.00(10)
Crystal system	monoclinic	monoclinic
Space group	$P2_1/c$	$P2_1$
a/Å	9.2624(3)	11.1911(4)
b/Å	15.2033(3)	7.3756(3)
c/Å	11.7020(3)	12.2859(5)
α/°	90	90
β/°	111.823(3)	104.786(4)
γ/°	90	90
Volume/Å^3	1529.77(8)	980.51(7)
Z	4	2
ρ_{calc}g/cm^3	1.434	1.384
μ/mm^{-1}	2.305	2.911
F(000)	696	432
Crystal size/mm^3	0.08 × 0.06 × 0.04	0.05 × 0.03 × 0.02
Radiation	Cu Kα (λ = 1.54184)	Cu Kα (λ = 1.54184)
2Θ range for data collection/°	10.008 to 134.972	7.442 to 134.79
Index ranges	$-11 \leq h \leq 10$, $-12 \leq k \leq 18$, $-14 \leq l \leq 14$	$-13 \leq h \leq 13$, $-5 \leq k \leq 8$, $-14 \leq l \leq 14$
Reflections collected	10816	4474
Independent reflections	2749 [R_{int} = 0.0327, R_{sigma} = 0.0336]	2343 [R_{int} = 0.0216, R_{sigma} = 0.0338]
Data/restraints/parameters	2749/8/233	2343/137/343
Goodness-of-fit on F^2	1.049	1.055
Final R indexes [I>=2σ (I)]	R_1 = 0.0450, wR_2 = 0.1180	R_1 = 0.0485, wR_2 = 0.1172
Final R indexes [all data]	R_1 = 0.0490, wR_2 = 0.1211	R_1 = 0.0615, wR_2 = 0.1267
Largest diff. peak/hole / e Å^{-3}	0.67/-0.37	0.32/-0.31

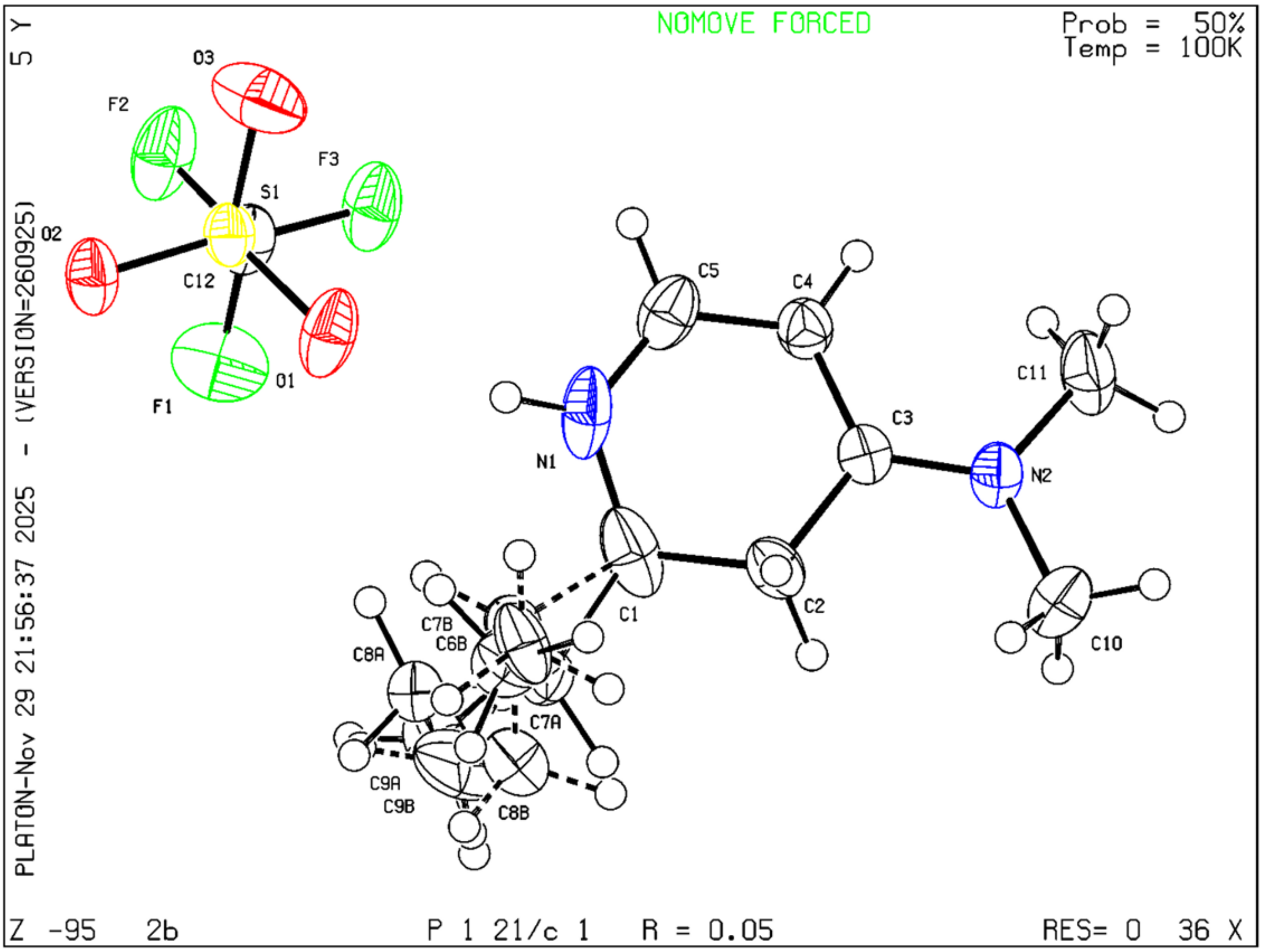

Figure S10. Molecular structure of **2b·OTf** (50% probability level)

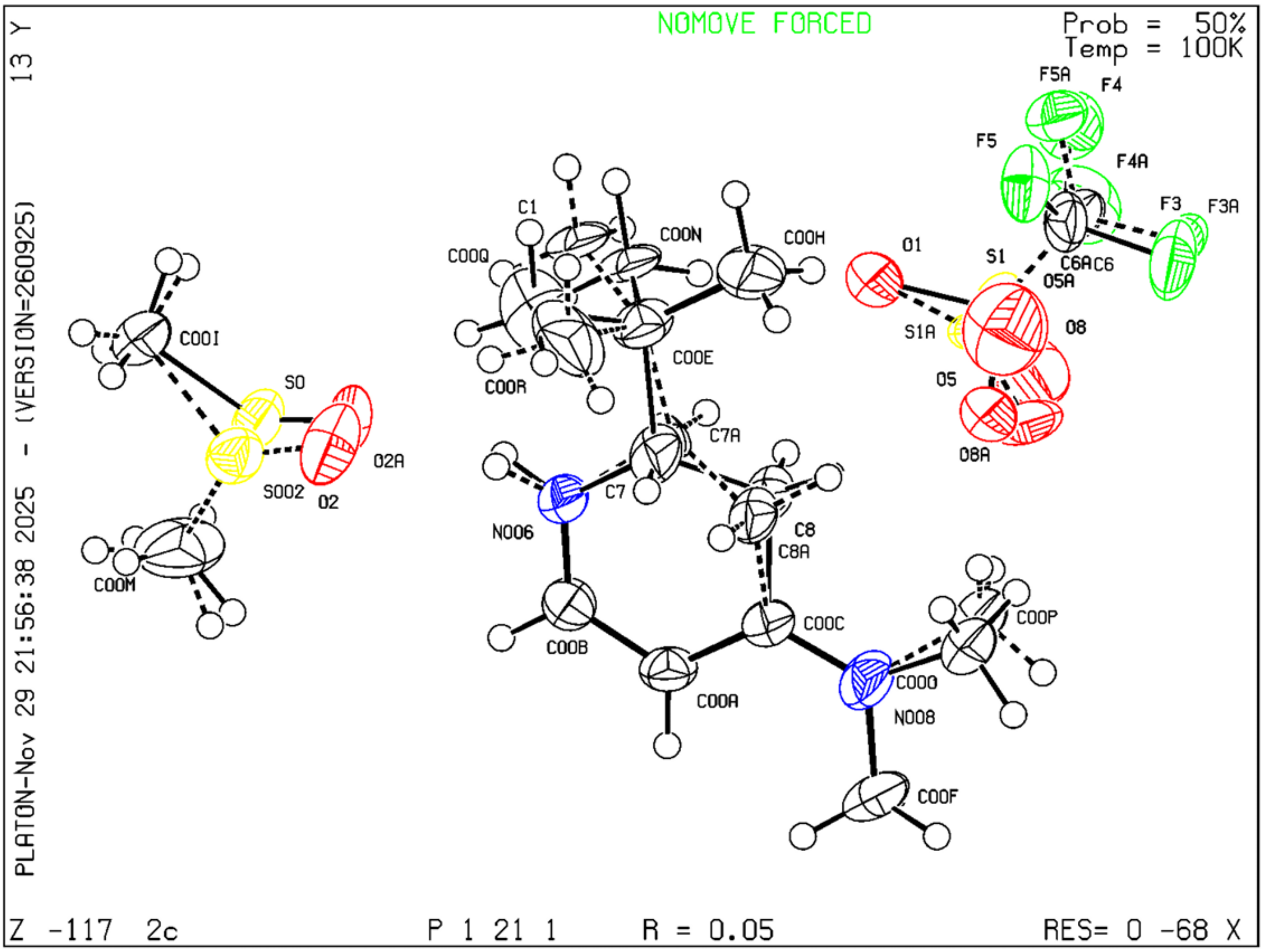

Figure S11. Molecular structure of **2c·OTf·DMSO** (50% probability level)

NMR spectra

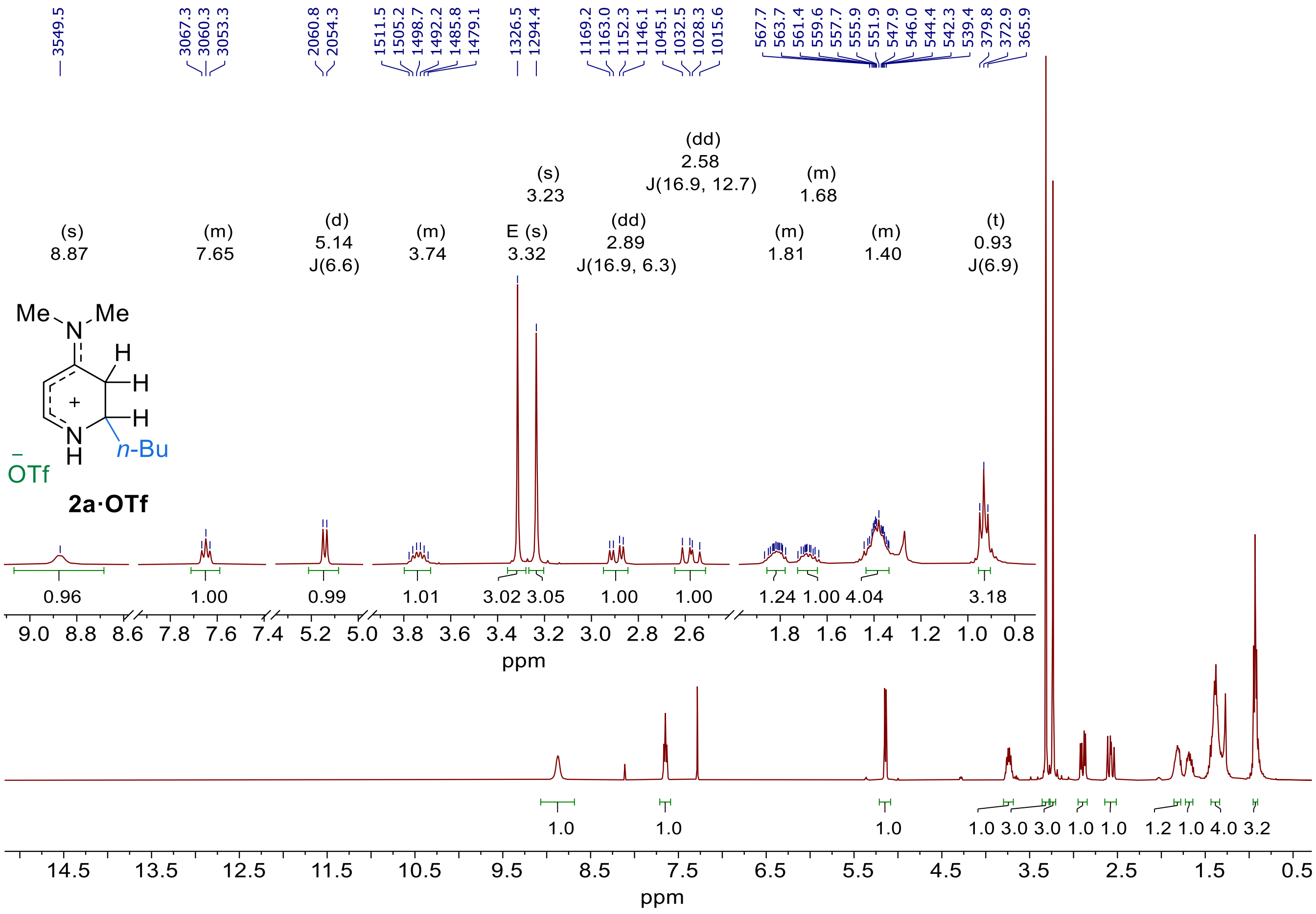

Figure S12. ^{1}H NMR spectrum of compound **2a·OTf** (400 MHz, $CDCl_3$)

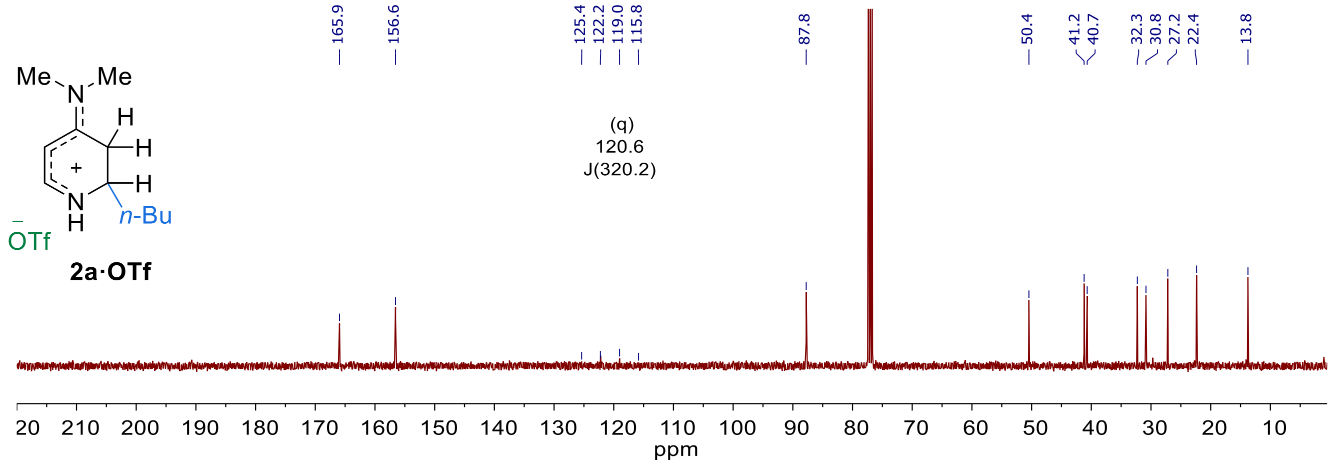

Figure S13. $^{13}C\{^{1}H\}$ NMR spectrum of compound **2a·OTf** (100 MHz, $CDCl_3$)

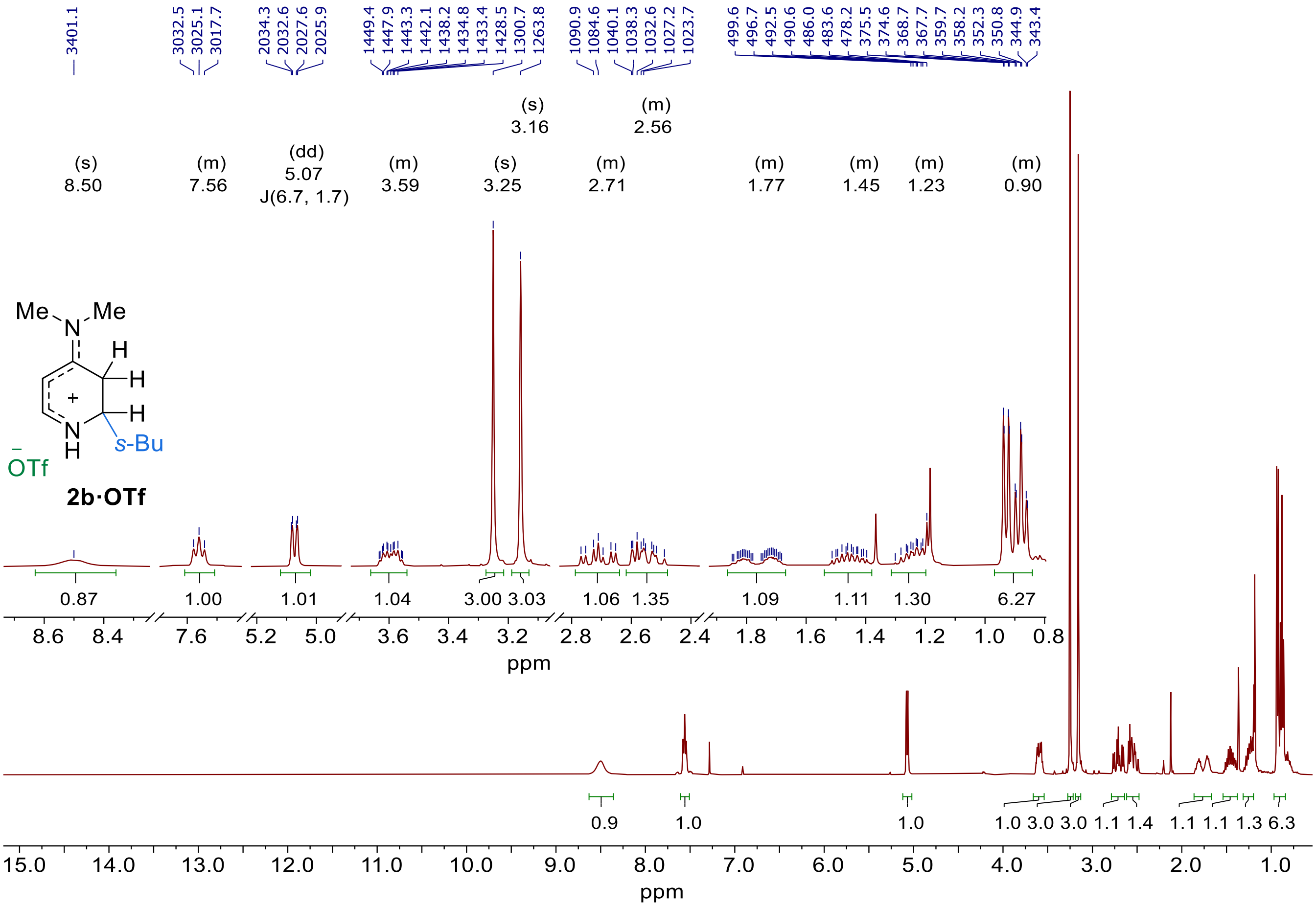

Figure S14. ^{1}H NMR spectrum of compound **2b·OTf** (400 MHz, $CDCl_3$)

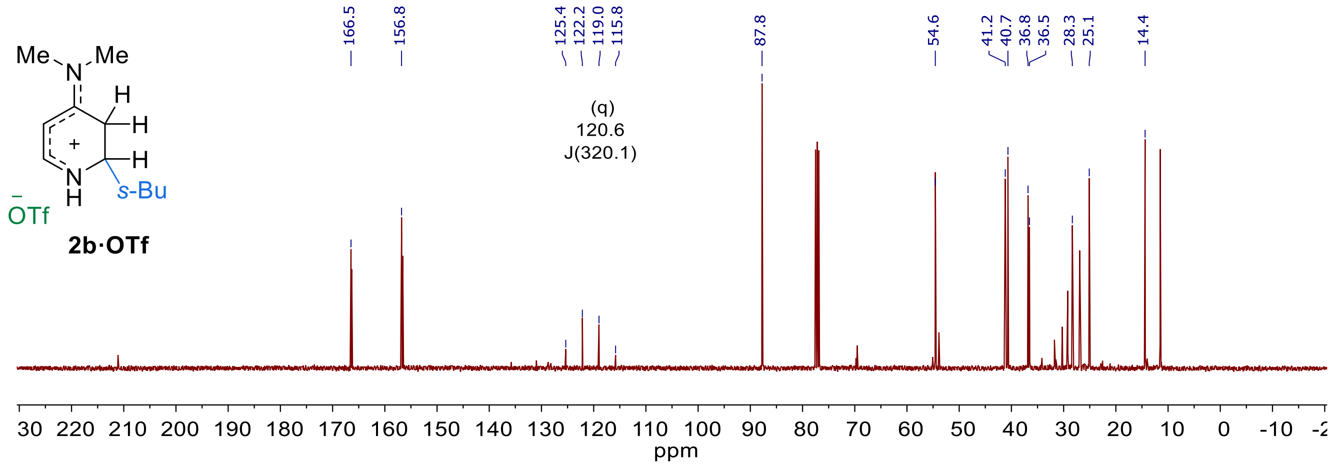

Figure S15. $^{13}C\{^{1}H\}$ NMR spectrum of compound **2b·OTf** (100 MHz, $CDCl_3$)

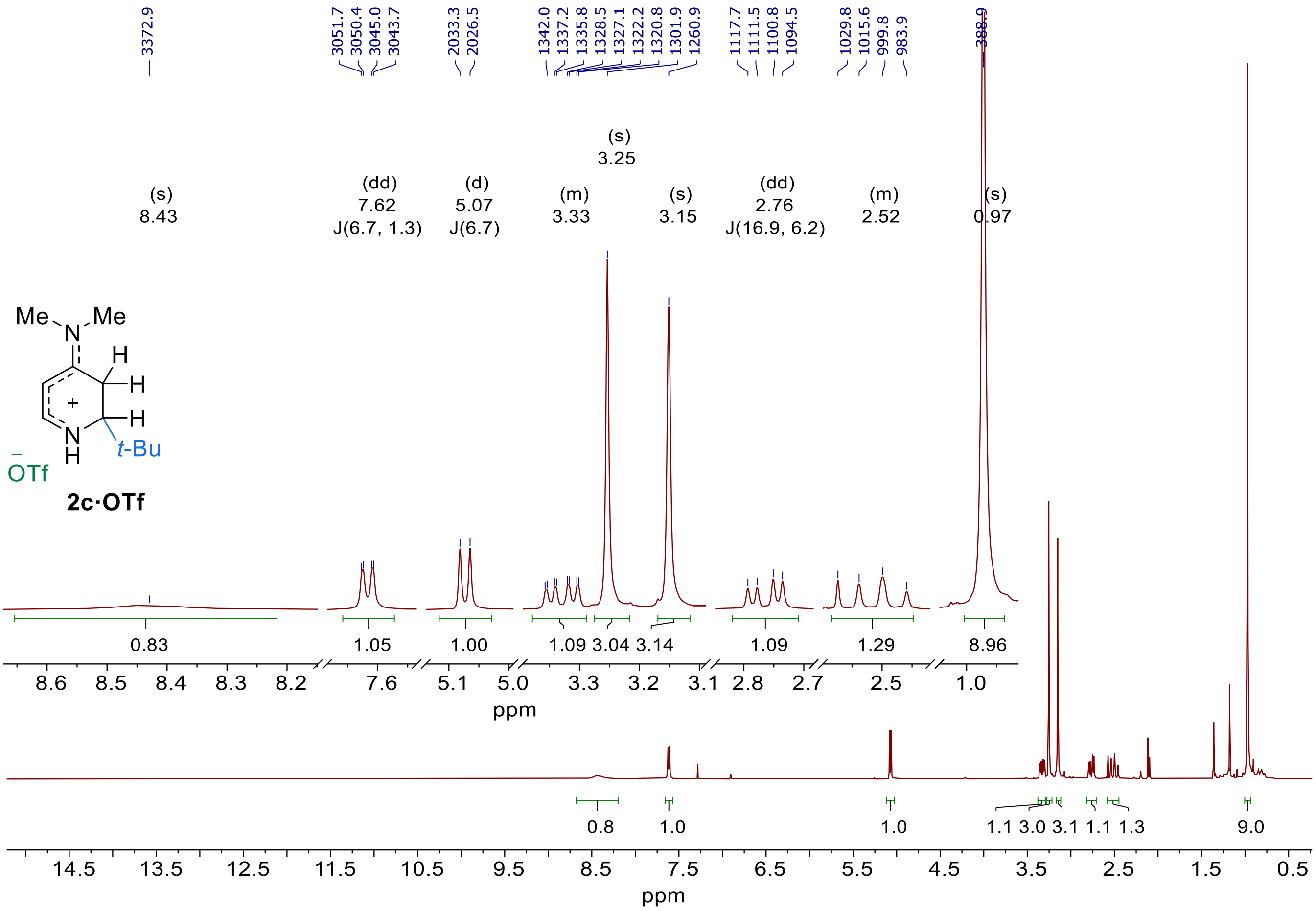

Figure S16. ^{1}H NMR spectrum of compound **2c·OTf** (400 MHz, $CDCl_3$)

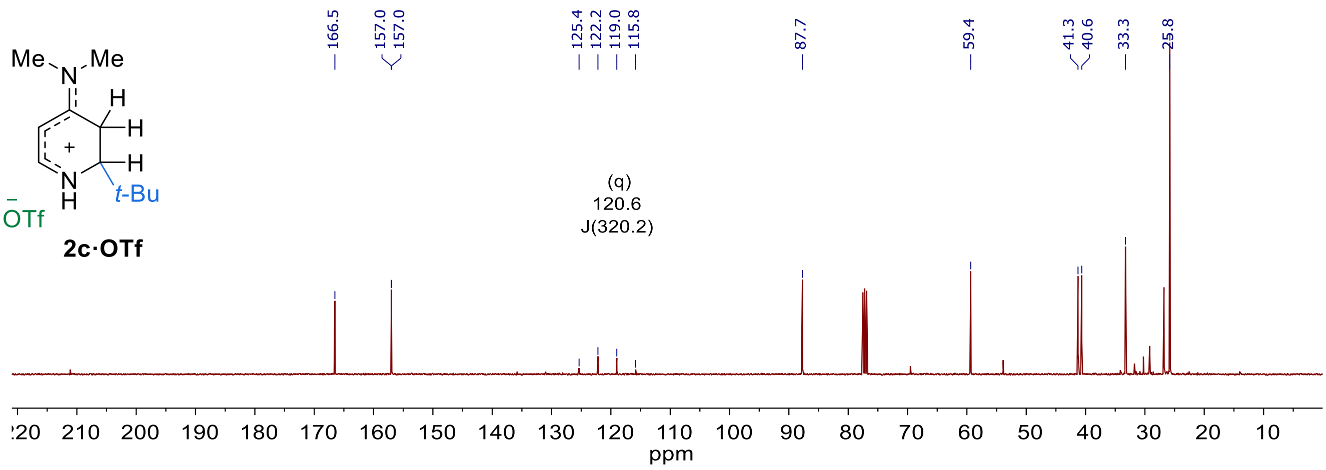

Figure S17. ^{13}C NMR spectrum of compound **2c·OTf** (100 MHz, $CDCl_3$)

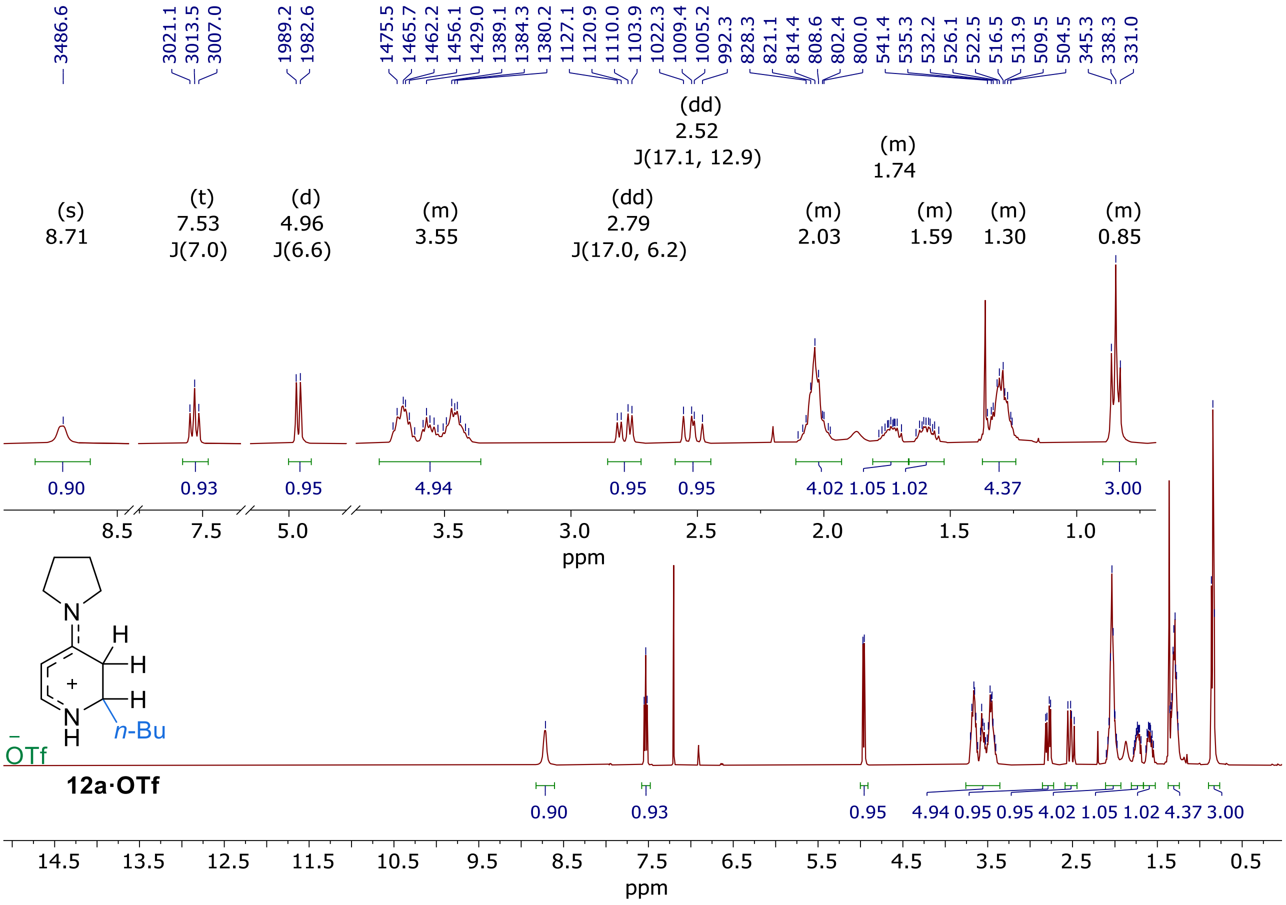

Figure S18. ^{1}H NMR spectrum of compound **12a·OTf** (400 MHz, $CDCl_3$)

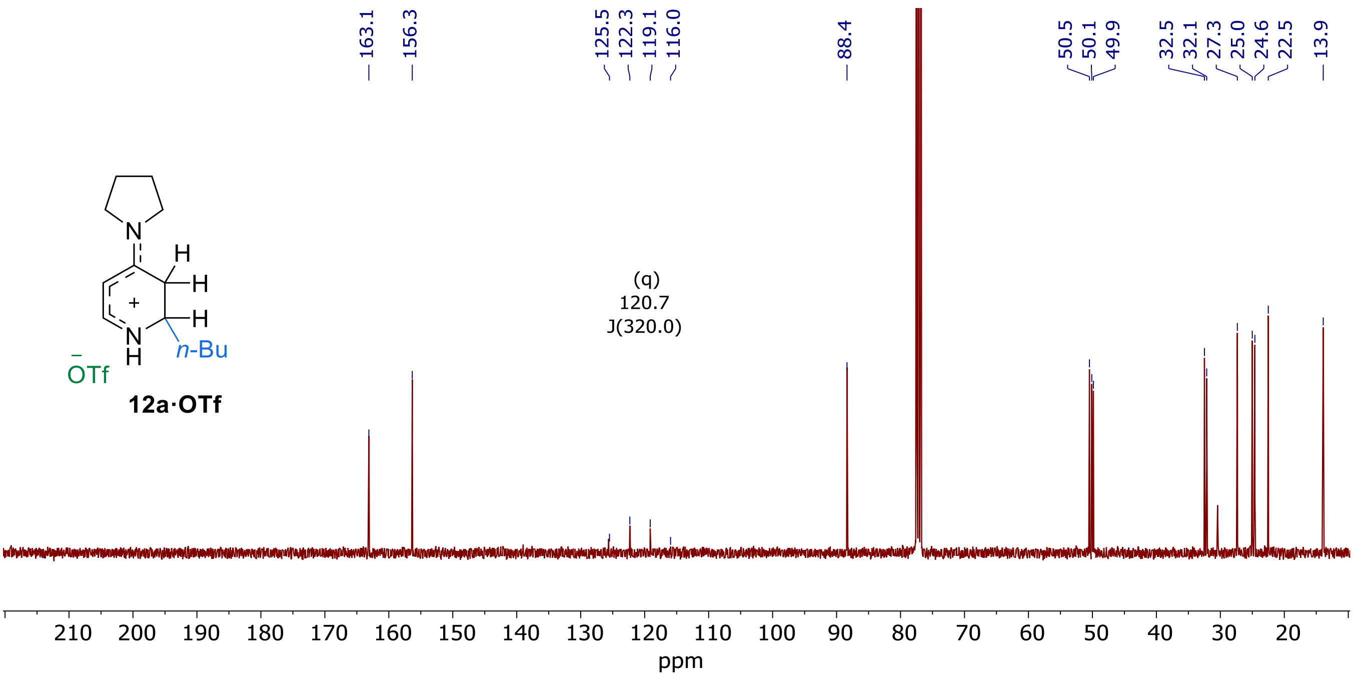

Figure S19. ^{13}C NMR spectrum of compound **12a·OTf** (100 MHz, $CDCl_3$)

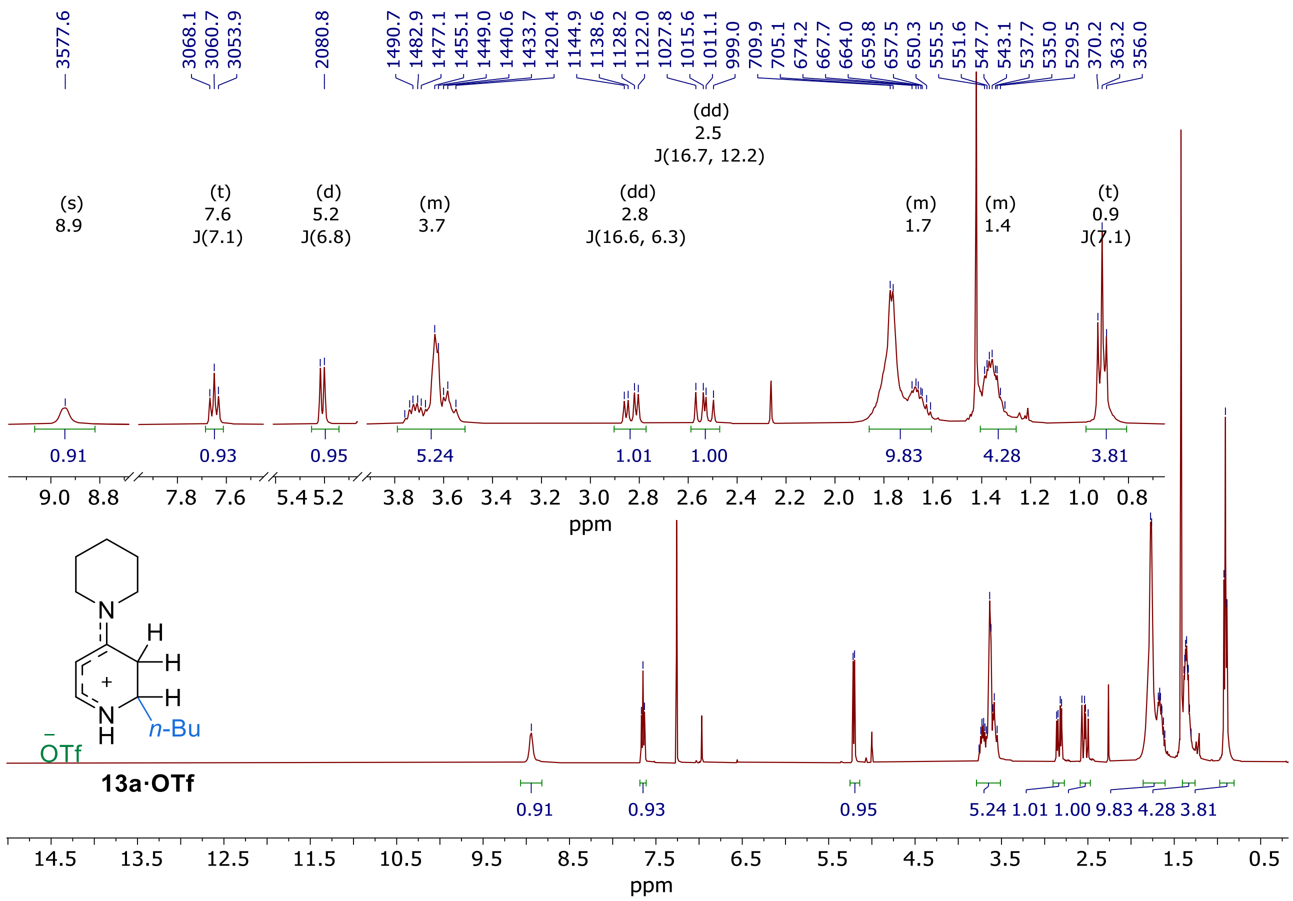

Figure S20. ^{1}H NMR spectrum of compound **13a·OTf** (400 MHz, $CDCl_3$)

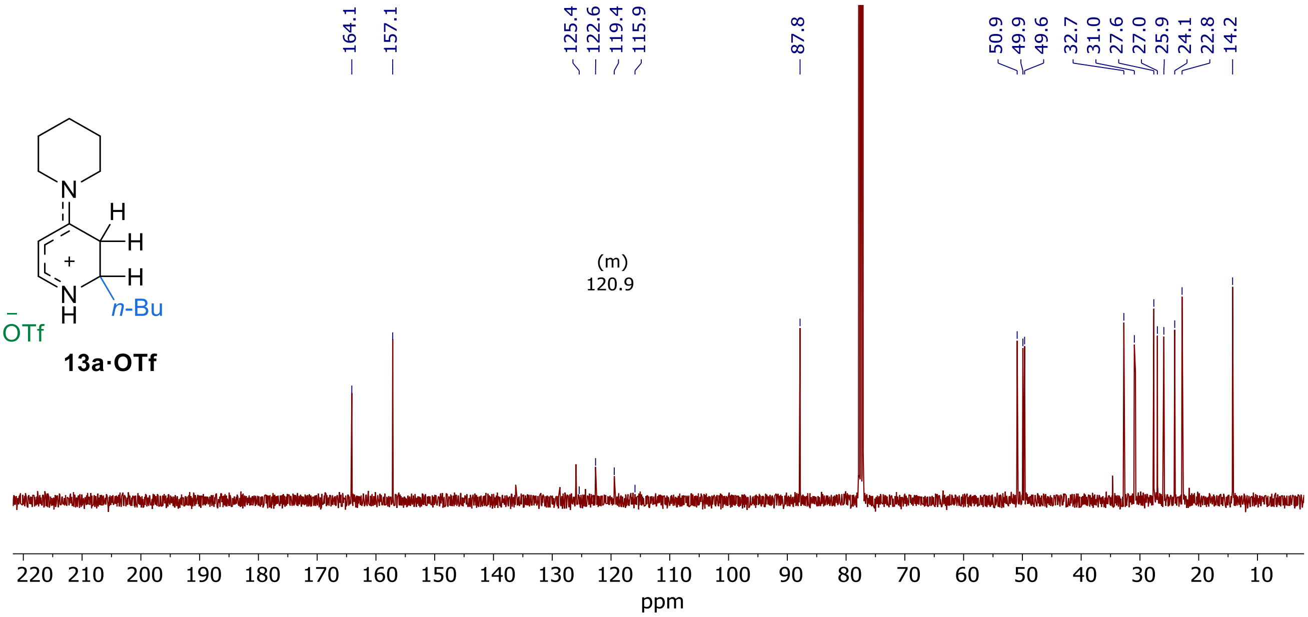

Figure S21. ^{13}C NMR spectrum of compound **13a·OTf** (100 MHz, $CDCl_3$)

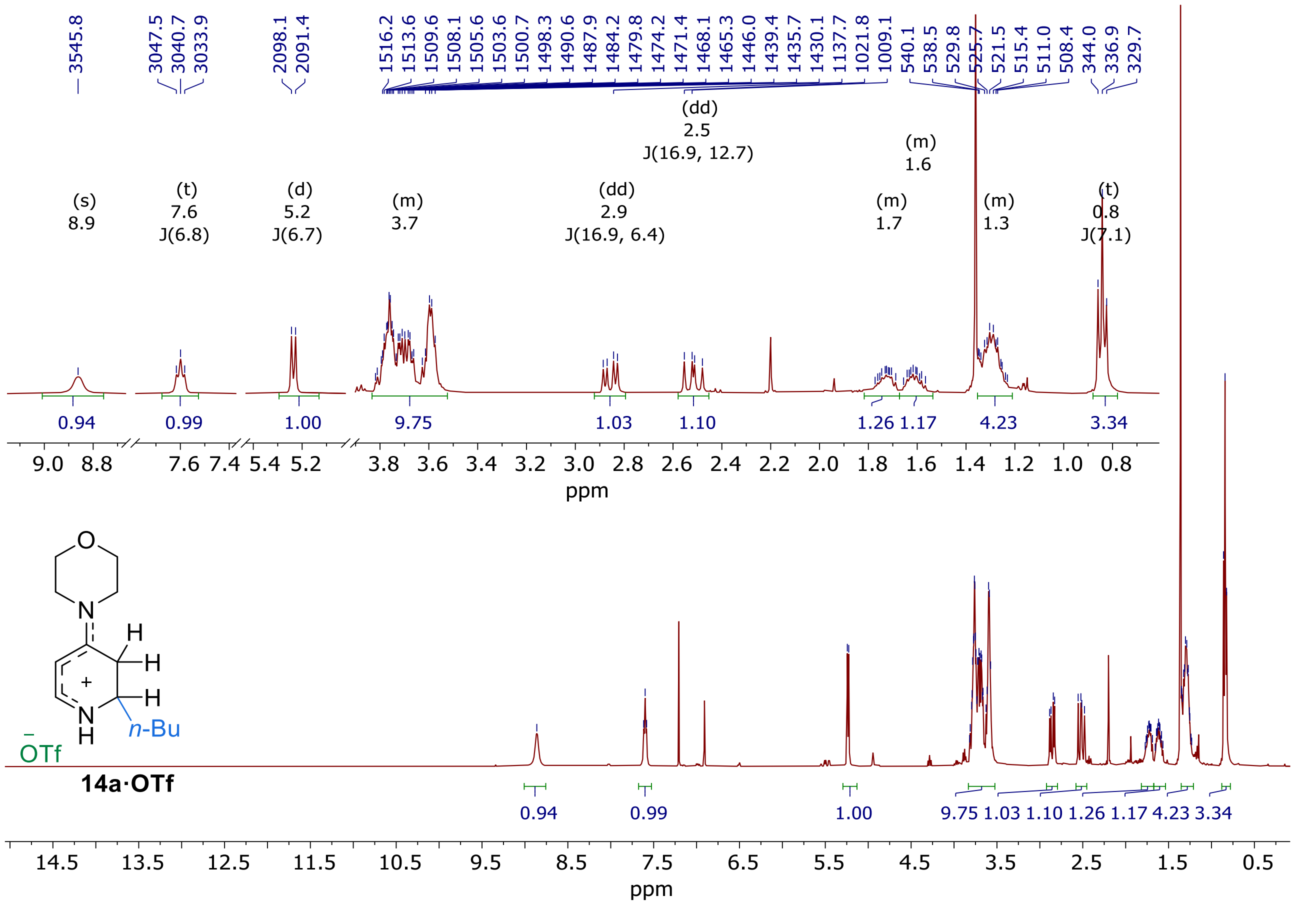

Figure S22. ^{1}H NMR spectrum of compound **14a·OTf** (400 MHz, $CDCl_3$)

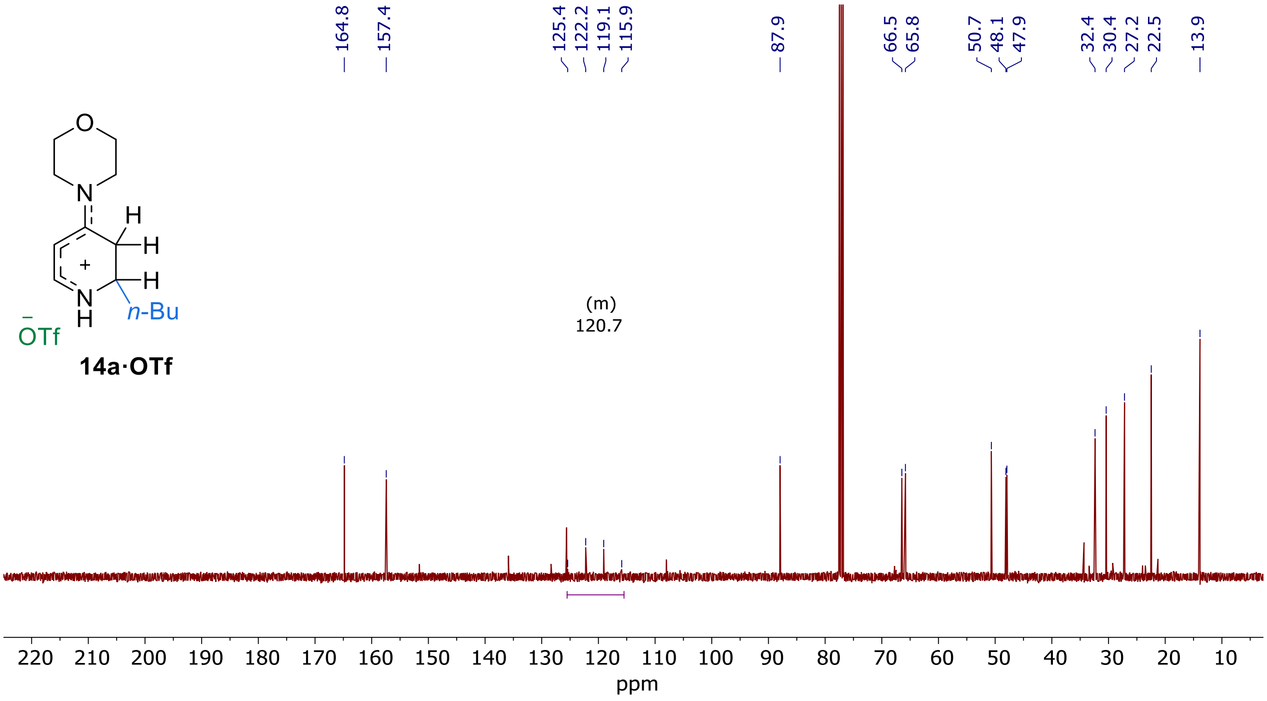

Figure S23. ^{13}C NMR spectrum of compound **14a·OTf** (100 MHz, $CDCl_3$)

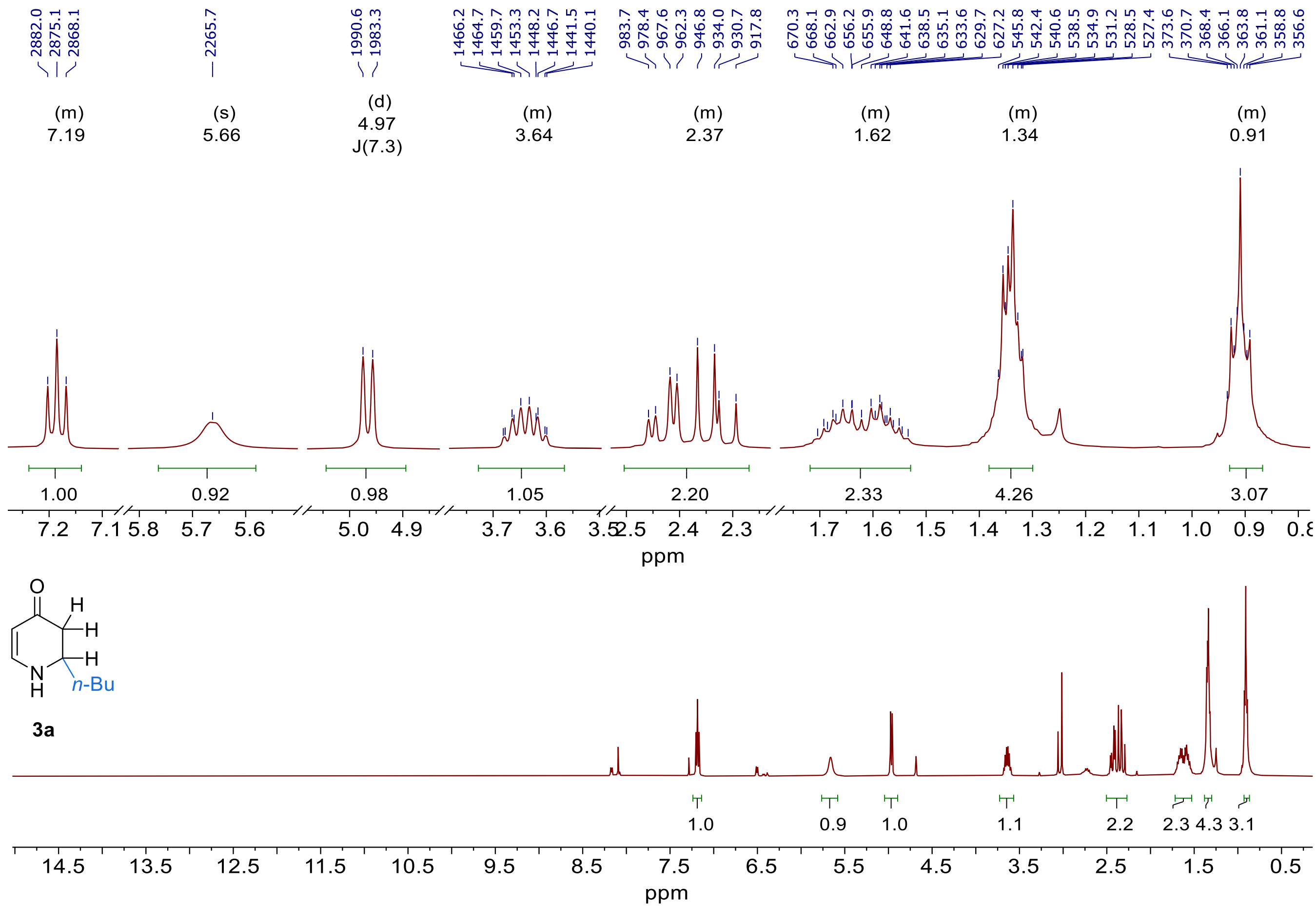

Figure S24. ^{1}H NMR spectrum of compound **3a** (400 MHz, $CDCl_3$)

References

(1) Frisch, M. J.; Trucks, G. W.; Schlegel, H. B.; Scuseria, G. E.; Robb, M. a.; Cheeseman, J. R.; Scalmani, G.; Barone, V.; Petersson, G. a.; Nakatsuji, H.; Li, X.; Caricato, M.; Marenich, a. V.; Bloino, J.; Janesko, B. G.; Gomperts, R.; Mennucci, B.; Hratchian, H. P.; Ortiz, J. V.; Izmaylov, a. F.; Sonnenberg, J. L.; Williams; Ding, F.; Lipparini, F.; Egidi, F.; Goings, J.; Peng, B.; Petrone, A.; Henderson, T.; Ranasinghe, D.; Zakrzewski, V. G.; Gao, J.; Rega, N.; Zheng, G.; Liang, W.; Hada, M.; Ehara, M.; Toyota, K.; Fukuda, R.; Hasegawa, J.; Ishida, M.; Nakajima, T.; Honda, Y.; Kitao, O.; Nakai, H.; Vreven, T.; Throssell, K.; Montgomery Jr., J. a.; Peralta, J. E.; Ogliaro, F.; Bearpark, M. J.; Heyd, J. J.; Brothers, E. N.; Kudin, K. N.; Staroverov, V. N.; Keith, T. a.; Kobayashi, R.; Normand, J.; Raghavachari, K.; Rendell, a. P.; Burant, J. C.; Iyengar, S. S.; Tomasi, J.; Cossi, M.; Millam, J. M.; Klene, M.; Adamo, C.; Cammi, R.; Ochterski, J. W.; Martin, R. L.; Morokuma, K.; Farkas, O.; Foresman, J. B.; Fox, D. J. Gaussian 16. Wallingford CT 2016, p Gaussian 16, Revision C.01, Gaussian, Inc., Wallin.

(2) Becke, A. D. Density-Functional Thermochemistry. I. The Effect of the Exchange Only Gradient Correction. *J. Chem. Phys.* **1992**, *96* (3), 2155–2160. https://doi.org/10.1063/1.462066.

(3) Lee, C.; Yang, W.; Parr, R. G. Development of the Colle-Salvetti Correlation-Energy Formula into a Functional of the Electron Density. *Phys. Rev. B* **1988**, *37* (2), 785–789. https://doi.org/10.1103/PhysRevB.37.785.

(4) Zhao, Y.; Truhlar, D. G. Design of Density Functionals That Are Broadly Accurate for Thermochemistry, Thermochemical Kinetics, and Nonbonded Interactions. *J. Phys. Chem. A* **2005**, *109* (25), 5656–5667. https://doi.org/10.1021/jp050536c.

(5) Hellweg, A.; Rappoport, D. Development of New Auxiliary Basis Functions of the Karlsruhe Segmented Contracted Basis Sets Including Diffuse Basis Functions (Def2-SVPD, Def2-TZVPPD, and Def2-QVPPD) for RI-MP2 and RI-CC Calculations. *Phys. Chem. Chem. Phys.* **2015**, *17* (2), 1010–1017. https://doi.org/10.1039/C4CP04286G.

(6) Grimme, S.; Ehrlich, S.; Goerigk, L. Effect of the Damping Function in Dispersion Corrected Density Functional Theory. *J. Comput. Chem.* **2011**, *32* (7), 1456–1465. https://doi.org/10.1002/jcc.21759.

(7) Lu, T.; Chen, F. Multiwfn: A Multifunctional Wavefunction Analyzer. *J. Comput. Chem.* **2012**, *33* (5), 580–592. https://doi.org/10.1002/jcc.22885.

(8) Savin, A.; Nesper, R.; Wengert, S.; Fässler, T. F. ELF: The Electron Localization Function. *Angew. Chemie Int. Ed. English* **1997**, *36* (17), 1808–1832. https://doi.org/10.1002/anie.199718081.

(9) Zhang, J. <scp>libreta</Scp> : Computerized Optimization and Code Synthesis for Electron Repulsion Integral Evaluation. *J. Chem. Theory Comput.* **2018**, *14* (2), 572–587. https://doi.org/10.1021/acs.jctc.7b00788.

(10) Sheldrick, G. M. {\it SHELXT} {--} Integrated Space-Group and Crystal-Structure Determination. *Acta Crystallogr. Sect. A* **2015**, *71* (1), 3–8. https://doi.org/10.1107/S2053273314026370.

(11) Sheldrick, G. M. Crystal Structure Refinement with {\it SHELXL}. *Acta Crystallogr. Sect. C* **2015**, *71* (1), 3–8. https://doi.org/10.1107/S2053229614024218.

(12) Dolomanov, O. V; Bourhis, L. J.; Gildea, R. J.; Howard, J. A. K.; Puschmann, H. {\it OLEX2}: A Complete Structure Solution, Refinement and Analysis Program. *J. Appl. Crystallogr.* **2009**, *42* (2), 339–341. https://doi.org/10.1107/S0021889808042726.

(13) Sax, M.; Berning, S.; Wünsch, B. One Pot Solid Phase Synthesis of 2-Substituted 2,3-Dihydropyridin-4(1H)- Ones on Rinkamide-Resin. *Tetrahedron* **2005**, *61* (1), 205–211. https://doi.org/10.1016/j.tet.2004.10.031.
